# The application of transcriptomic data in the authentication of beef derived from contrasting production systems

**DOI:** 10.1186/s12864-016-2851-7

**Published:** 2016-09-21

**Authors:** Torres Sweeney, Alex Lejeune, Aidan P. Moloney, Frank J. Monahan, Paul Mc Gettigan, Gerard Downey, Stephen D. E. Park, Marion T. Ryan

**Affiliations:** 1 UCD College of Agriculture, Food Science and Veterinary Medicine, University College Dublin, Belfield, Dublin 4, Ireland; 2 Teagasc, Animal and Grassland Research and Innovation Centre, Grange, Dunsany, Co. Meath Ireland; 3 Ashtown Food Research Centre, Dunsinea, Co. Dublin Ireland

**Keywords:** Beef authentication, β-oxidation, Epigenetics, Fatty acid, Concentrates, Pasture, PPAR, Transcriptome

## Abstract

**Background:**

Differences between cattle production systems can influence the nutritional and sensory characteristics of beef, in particular its fatty acid (FA) composition. As beef products derived from pasture-based systems can demand a higher premium from consumers, there is a need to understand the biological characteristics of pasture produced meat and subsequently to develop methods of authentication for these products. Here, we describe an approach to authentication that focuses on differences in the transcriptomic profile of muscle from animals finished in different systems of production of practical relevance to the Irish beef industry. The objectives of this study were to identify a panel of differentially expressed (DE) genes/networks in the muscle of cattle raised outdoors on pasture compared to animals raised indoors on a concentrate based diet and to subsequently identify an optimum panel which can classify the meat based on a production system.

**Results:**

A comparison of the muscle transcriptome of outdoor/pasture-fed and Indoor/concentrate-fed cattle resulted in the identification of 26 DE genes. Functional analysis of these genes identified two significant networks (1: Energy Production, Lipid Metabolism, Small Molecule Biochemistry; and 2: Lipid Metabolism, Molecular Transport, Small Molecule Biochemistry), both of which are involved in FA metabolism. The expression of selected up-regulated genes in the outdoor/pasture-fed animals correlated positively with the total n-3 FA content of the muscle. The pathway and network analysis of the DE genes indicate that peroxisome proliferator-activated receptor (PPAR) and FYN/AMPK could be implicit in the regulation of these alterations to the lipid profile. In terms of authentication, the expression profile of three DE genes (*ALAD, EIF4EBP1 and NPNT)* could almost completely separate the samples based on production system (95 % authentication for animals on pasture-based and 100 % for animals on concentrate- based diet) in this context.

**Conclusions:**

The majority of DE genes between muscle of the outdoor/pasture-fed and concentrate-fed cattle were related to lipid metabolism and in particular β-oxidation. In this experiment the combined expression profiles of *ALAD, EIF4EBP1* and *NPNT* were optimal in classifying the muscle transcriptome based on production system. Given the overall lack of comparable studies and variable concordance with those that do exist, the use of transcriptomic data in authenticating production systems requires more exploration across a range of contexts and breeds.

**Electronic supplementary material:**

The online version of this article (doi:10.1186/s12864-016-2851-7) contains supplementary material, which is available to authorized users.

## Background

The feeding regimen used in beef production influence both the economics of beef production and the nutritional quality of beef. In temperate regions, grazed and conserved grass systems are considered less expensive options than concentrate feeds [1–3]. Human health advantages associated with the consumption of beef reared on pasture have been proposed, particularly relating to the supply of essential Fatty Acids (FA) [4, 5], with beef from pasture systems considered nutritionally superior to beef from concentrate-based systems, due to its greater conjugated linoleic acid content [6], n-3/n-6 FA ratio [7] and vitamin E content [8]. Dietary intakes of total n-3 PUFAs, as well as plasma and platelet concentrations of LC n-3 PUFA, were significantly higher in subjects who consumed red meat from pasture-fed animals compared with those who consumed red meat from concentrate-fed animals [9]. In addition, there is some evidence that regular outdoor exercise has positive effects on the health and welfare status of cattle [10, 11].

Clearly, authentication of beef would be of economic benefit as it would increase consumer confidence in the characteristics, source and subsequent pricing of meat based on their production system. There have been a number of approaches used to authenticate meat products in a range of species including carotenoid content and color measurements [12], triacylglycerol profiles [13], volatile hydrocarbons [14], stable isotope ratios [15], metabolomic data [16] or combinations of these variables [17]. In addition, a number of studies have explored the possibility of utilising functional genomics, in particular, transcriptome and proteome profiling, to discriminate between animals reared under different production systems, as it is hypothesised that different diets will alter the expression of genes involved in fat/muscle metabolism [18–20].

In order to achieve the ultimate goal of finding both individual biomarkers and/or molecular signatures with which to authenticate meat products as having originated from pasture-based systems, it is necessary to identify a robust and reproducible panel of genes which are differentially expressed (DE) between muscle of cattle raised in pasture and concentrate-based production systems. Therefore, the objectives of this study were: 1) to identify a panel of DE genes in the muscle of cattle raised outdoors on pasture compared to animals raised indoors on a concentrate-based diet; 2) to identify relationships or functional commonalities between these genes, and 3) to subsequently identify an optimum panel which will classify the meat based on a pasture-based production system.

## Results

### DE genes between outdoor-pasture and indoor-concentrate-fed animals

Thirty-two probe sets were highlighted as DE (*P* < 0.05) between the outdoor/pasture-fed and indoor/concentrate-fed cattle. These probe sets corresponded to 26 genes, of which 16 were up-regulated and 10 down-regulated in the muscle of the outdoor/pasture-fed and indoor/concentrate-fed cattle, respectively (Table 1).

**Table 1:** Overview of DE genes including probe IDs, gene symbol, accession number, description, log fold change and significance

	Annotation probe ID	Gene symbol	Accession number	Gene description^a^	Microarray	
					Log FC	Adj *p*-value
Up-regulated in Pasture-fed group	Bt.21113.1.S1_a_at	*CPT1B*	NM_001034349	carnitine palmitoyltransferase 1B (muscle)	−0.65	0.02
	Bt.17513.1.A1_at	*PLIN5*	NM_001101136	perilipin 5	−1.11	0.03
	Bt.2359.1.A1_at	*FYN*	NM_001077972	oncogene related to SRC, FGR, YES	−0.79	0.01
	Bt.19423.2.S1_at	*ABCA1*	NM_001024693	ATP-binding cassette, sub-family A (ABC1), member 1	−1.12	0.05
	Bt.16916.3.S1_at	*KLF11*	XM_868832	Kruppel-like factor 11	−1.12	0.002
	Bt.22869.1.S2_at	*FABP5*	NM_174315	fatty acid binding protein 5 (psoriasis-associated)	−0.76	0.02
	Bt.1739.2.S1_at	*FZD4*	NM_001077972	frizzled family receptor 4	−0.62	0.03
	Bt.4757.1.S1_at	*ARHGDIB*	NM_175797	Rho GDP dissociation inhibitor beta	−0.44	0.04
	Bt.5389.1.S1_at	*EIF4EBP1*	NM_001077893	eukaryotic translation initiation factor 4E binding protein 1	−0.79	0.05
	Bt.9585.1.S1_at	*ALAD*	NM_001014895	aminolevulinate dehydratase	−1.03	0.03
	Bt.1035.1.S1_a_at	*FCGRT*	NM_176657	Fc fragment of IgG, receptor, transporter, alpha	−0.96	0.01
	Bt.6936.1.S1_at	*CCL14*	NM_001046585	chemokine (C-C motif) ligand 14	−0.62	0.03
	Bt.19795.1.S1_at	*BREH1*	NM_001012287	retinyl ester hydrolase type 1 precursor	−0.57	0.03
	Bt.6434.2.S1_at	*RNF149*	XM_582694	ring finger protein 149	−0.52	0.03
	Bt.19850.2.S1_at	*ACSL3*	XM_001787476	long-chain-fatty-acid--CoA ligase 3	−0.44	0.03
	Bt.26962.1.S1_at	*GPIHBP1*	XM_590408	glycosylphosphatidylinositol anchored high density lipoprotein binding protein 1	−0.41	0.05
Down-regulated in Pasture-fed group	Bt.6394.1.A1_at	*STK40*	NM_001075727	serine/threonine kinase 40	0.42	0.03
	Bt.2392.1.S1_at	*ST6GALNAC4*	NM_205791.1	ST6 (alpha-N-acetyl-neuraminyl-2,3-beta-galactosyl-1,3)-N-acetylgalactosaminide alpha-2,6-sialyltransferase 4	0.71	0.03
	Bt.20458.1.S1_at	*MAP7D1*	XM_589552	MAP7 domain containing 1	0.60	0.02
	Bt.7393.1.S1_at	*NPNT1*	XM_002688090.3	nephronectin	1.07	0.03
	Bt.11038.1.S1_at	*TULP1*	XM_865044	tubby like protein 1	0.46	0.002
	Bt.3562.1.S1_at	*LDLR*	NM_001166530	low density lipoprotein receptor	0.46	0.03
	Bt.23212.1.S1_at	*MSMO1*	NM_001098863.1	methylsterol monooxygenase 1	0.34	0.05
	Bt.4688.1.S1_a_at	*TPCN1*	XM_588037	two pore segment channel 1	0.36	0.03
	Bt.13526.1.S1_at	*PDP2*	NM_001206353	pyruvate dehyrogenase phosphatase catalytic subunit	0.40	0.03
	Bt.16265.1.S1_at	*EML1*	XM_590509	echinoderm microtubule associated protein like 1	0.42	0.03

^a^Gene descriptions were obtained from GeneCards and the NCBI Entrez Gene database

### Systems biology analysis of differentially expressed genes

**Fig. 1:**
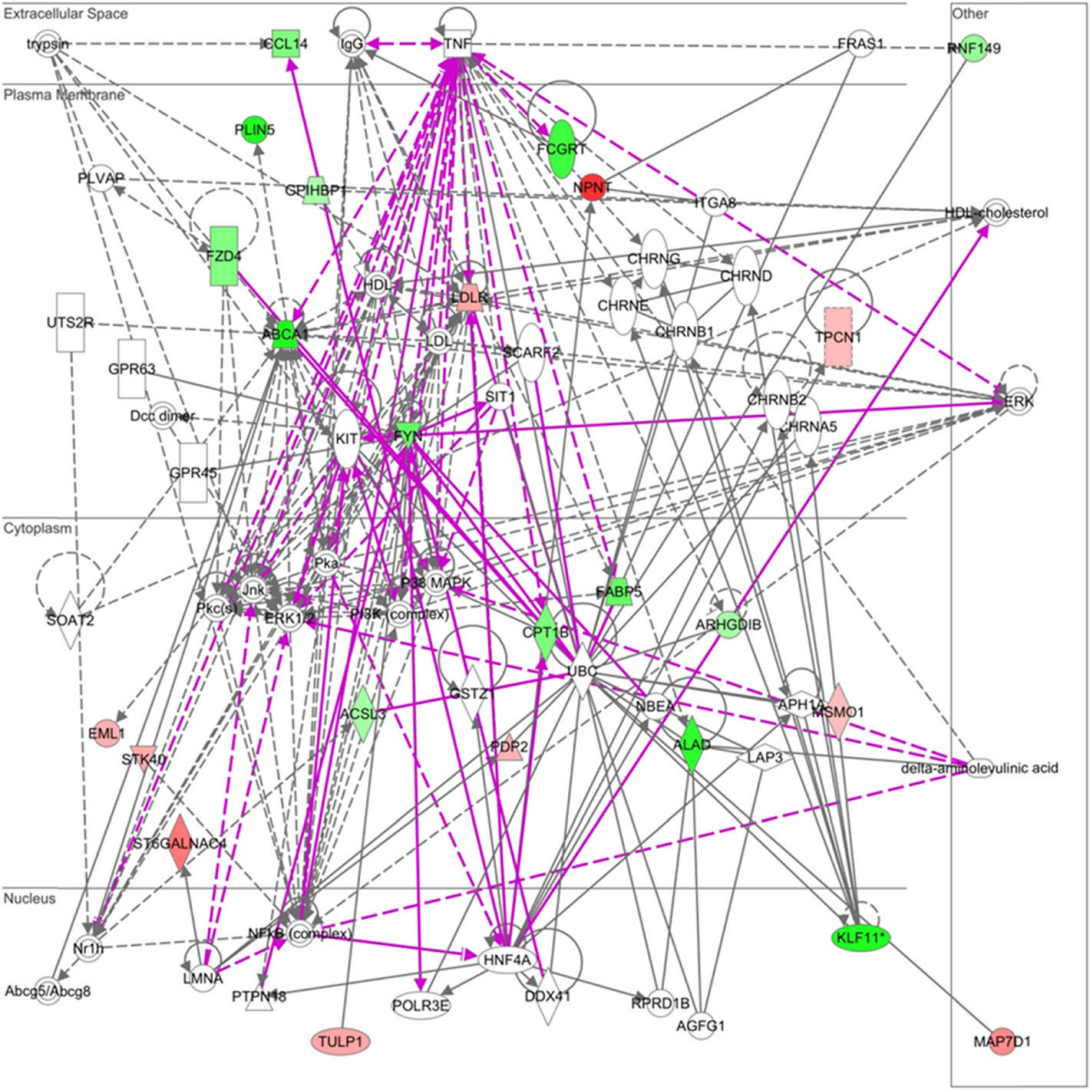
Merged top two significant networks highlighting direction of DE genes i.e. up (red) and down (green) in outdoor-pasture-fed animals

Two significant networks were identified using Ingenuity Pathway Analysis (IPA), and these are presented as a merged network in Fig. 1. The main functions of these two networks were: 1) Energy Production, Lipid Metabolism, Small Molecule Biochemistry (Score = 30); and, 2) Lipid Metabolism, Molecular Transport, Small Molecule Biochemistry (Score =24).

Five of the genes that were up-regulated in the outdoor/pasture-fed (*CPT1B, ABCA1, FABP5, ACSL3* and *GPIHBP1*) and two of the genes that were down-regulated in the outdoor/pasture-fed group (*LDLR* and *MSMO1*) related to Gene Ontology (GO) terms relating to molecular function directly aligned to FA metabolism.

Two significant pathways were identified using the Database for Annotation, Visualization and Integrated Discovery (DAVID) pathway analysis: 1) PPAR signaling pathway (*CPTB1, FAPB5* and *ACSL3*); and 2) FA degradation (*CPTB1* and *ACSL3*), these are presented in Additional file 1: Figure S1 and S2. Based on subsequent literature searches for all 26 genes, several additional genes are associated with FA metabolism and intramuscular Fat (IMF) (Table 2). These literature searches confirmed that many of the DE genes are linked to key regulators of adipogenesis, such as SCREB, C/EBP. In particular many of the DE genes were cited as downstream targets PPAR including, *KLF11* [21], *CCL14* [22]*, PLIN5* [23], *ABCA1* [21, 24], *FYN* [25], *ST6GALNAC* [26], *LDLR* [27] and *FCGRT* [28]. These associations are summarised in Table 2.

**Table 2:** Outline of DE genes which are relevant to fatty acid metabolism based on Gene Ontogeny (GO) biological function and literature searches

Gene Symbol	GO Terms –Biological Process (selected terms related to lipid metabolism)	Description of gene function	Evidence relating the gene to meat quality, FA metabolism and regulation
*CPT1B*	fatty acid beta-oxidation - GO:0006635 carnitine shuttle - GO:0006853 long-chain fatty acid transport - GO:0015909 cellular lipid metabolic process - GO:0044255 regulation of fatty acid oxidation - GO:0046320	The *CPT1B* gene encodes an enzyme which is part of the carnitine shuttle, responsible for transferring long-chain FA across the barrier of the inner mitochondrial membrane to gain access to the enzymes of β-oxidation. *CPT1B* is the muscle isoform but it is also expressed in adipocytes.	Widely reported to be regulated by PPARδ in skeletal muscle [29]. Up-regulated in the muscle of grazing lambs relative to those reared indoors [30] and also in the muscle of Barrosã breed relative to the Alentegjana bulls (a breed which possesses higher fatty acid proportions within the subcutaneous adipose tissue) [31].
*PLIN 5*		Members of the perilipin family including *PLIN5,* coat intracellular lipid storage droplets and protecting them from lipolytic degradation [23].	Fatty acids reported to regulate *PLIN5* through the activation of PPARδ in muscle [32]. Muscles over-expressing *PLIN5* displayed a 44.8 % increase in fatty acid oxidation [33]. C/EBPα promotes transcription of PLIN5 in pigs [34]
*FYN*		*FYN*, encodes a tyrosine-specific kinase that belongs to the Src kinase family and is known to regulate cell proliferation and ion channel activity. The protein associates with the p85 subunit phosphatidylinositol 3-kinase and interacts with the FYN-binding protein [35].	*FYN* has been reported to regulate the activity of the adipogenic transcription factor STAT5a which subsequently initiates the expression of the master adipogenic transcription factors PPARγ and C/EBPα (Tse et al., 2013). PUFAs, arachidonic acid and eicosapentaenoic acid reported to inhibit Fyn palmitoylation, thereby blocking Fyn localisation to detergent-resistant membranes in T cells [36] Mice null for *Fyn* display reduced adipose mass associated with decreased adipocyte cell size. In parallel, a substantial reduction in fasting plasma glucose, insulin, triglycerides, and free fatty acids is evident concomitant with decreased intra-hepatocellular and intra-myocellular lipid accumulation [37].
*ABCA1*	phospholipid binding - GO:0005543 phospholipid transporter activity - GO:0005548 cholesterol binding - GO:0015485 cholesterol transporter activity - GO:0017127 apolipoprotein binding - GO:0034185 apolipoprotein A-I binding - GO:0034186 apolipoprotein A-I receptor activity - GO:0034188	*ABCA1* encodes a membrane-associated protein and is a member of the superfamily of ATP-binding cassette (ABC) transporters, which transport various molecules across extra- and intracellular membranes. *ABCA1* has been referred to as the gatekeeper of the reverse cholesterol transport pathway whereby excess cholesterol in peripheral tissues is transported to the liver for elimination from the body [38]	Reported to be regulated by PPARδ in cultured human muscle [24] and in human myotubes [21]. Expression of *ABCA1* was found to be correlated with beef traits in the *LD* muscle between 1 and 24 Months in Chinese Red Steppes [39] A SNP c27113G > A present in the *ABCA1* gene was reported to have significant associations with conjugated linoleic acid (CLA) in the muscle of a Waagyu x Limousin reference population [40] miR-758, miR-26 and miR-106b all reported to target *ABCA1* [41]
*KLF11*		*KLF11* is a ubiquitously expressed transcription factor which contains a Krüppel-like 3 zinc finger motif at the C-terminal end of the protein. *KLF11* binds GC rich Sp1-like sequences to regulate gene expression and inhibit cell proliferation [42]. Although *KLF11* was initially introduced as a TGF*-*β inducible gene, several studies have described its up-regulation by a range of growth factors, cytokines and hormones [43]	Reported to be regulated by PPARδ in human myotubes [21]
*FABP5*	fatty acid binding - GO:0005504 lipid binding - GO:0008289	*FABP5* is expressed in epidermal cells and adipocytes and belongs to a family of small, highly conserved, cytoplasmic proteins that bind long-chain fatty acids and have roles in fatty acid uptake, transport, and metabolism [44]	FABP5 shuttles ligands from the cytosol to the nuclear receptor PPAR thereby enhancing the transcriptional activity of the receptor [45] *FABP5* was differentially expressed between animals exhibiting divergent patterns of fatty acid composition in *LT* muscle [46] Protein expression correlated with subcutaneous fat thickness in British-continental steers on diets with differing levels of fat [47]
*EIF4EBP1*		Encodes a translation repressor proteins which interacts with eukaryotic translation initiation factor 4E (eIF4E), thereby repressing translation. It can be phosphorylated in response to various signals, including insulin.	Differentially expressed in LD muscle in Jinhua (high oxidative metabolism and adipogenesis) and Landrace (low oxidative metabolism and adipogenesis) pigs [48] Some evidence indicates that this class of translation repressor protein is inhibited by mTORC1 and important for the regulation of PPAR-γ and C/EBPs by mTORC1 [49]
*FZD4*		FZD4 is a member of the frizzled gene family of receptors. Most frizzled receptors are coupled to the beta-catenin canonical signalling pathway and may play a role as a positive regulator of the Wnt signalling pathway which plays a major role in differentiation and patterning during embryogenesis as well as regulating cell proliferation in adult tissues [50]	Expression of *FZD4* increases gradually during adipogenesis in human adipose tissue-derived stem cells and decreases in response to the anti-adipogenic agent isorhamnetin [51]
*FCGRT*			Up-regulated in hepatocytes cultured with the PPARδ agonist (KD3010) relative to the control [28]
*CCL14*		CCL14 is a chemokine that promotes trophoblast migration. CCL14 to be a potent promoter of breast cancer angiogenesis and metastasis [52]	Found to be induced by PPAR in primary human hepatocytes [22]
*ACSL3*	fatty acid biosynthetic process - GO:0006633 triglyceride biosynthetic process - GO:0019432 low-density lipoprotein particle assembly- GO:0034379 LC fatty-acyl-CoA biosynthetic process - GO:0035338 cellular lipid metabolic process - GO:0044255 long-chain fatty acid import- GO:0044539	The formation of acyl-CoA from fatty acid, ATP, and CoA is catalysed by acyl-CoA synthetase (ACS). This reaction an essential reaction in mammalian FA metabolism. Acyl-CoAs produced by ACS are mainly utilised both in the synthesis of cellular lipids and in degradation via the β-oxidation system for energy production. In addition to the production of acyl-CoA, ACS also facilitates the cellular uptake of long-chain fatty acids [53]. ACSL3 utilizes arachidonate and eicosapentaenoate most efficiently among the C_16_-C_20_ unsaturated fatty acids [54].	SNP associated with this gene was significantly associated with the percentages of oleic fatty acid and MUFA [55] Established as a PPARα target gene in bovine cell line [56] Reported to be regulated by PPARδ activation in human myotubes [21]
*GPIHBP1*	lipid transport - GO:0006869 cholesterol homeostasis - GO:0042632 positive regulation of lipoprotein lipase activity - GO:0051006 triglyceride homeostasis - GO:0070328	GPIHBP1 is a capillary endothelial cell protein that provides a platform for LPL-mediated processing of chylomicrons as transfection of mouse Gpihbp1 in CHO cells conferred the ability to bind LPL and chylomicrons [57]	Established as a protein of capillary endothelial cells and the principal binding site for LPL on endothelial cells, responsible for transporting LPL to the capillary lumen [58] Using transfected Chinese hamster ovary (CHO) it was demonstrated that mouse *Gpihbp1* bound to radiolabeled high density lipoprotein (HDL), and selectively bound the lipid component of HDL, but not cholesterol or protein [59]
*STK40*		*STK40* encodes a protein of 435 amino acid residues and contains a serine/threonine kinase domain. Transcript and protein levels of Stk40 were found to be up-regulated and maintained at high levels during the process of spontaneous embryoid-body (EB) formation [60]	In a GWAS > 1,000 human subjects lipoprotein measurements in a SNP rs3007220 in an intron within *STK40* was associated with HDL cholesterol concentrations [61]
*ST6GALNAC4*		The protein encoded by *ST6GALNAC4* is a type II membrane protein that catalyses the transfer of sialic acid from CMP-sialic acid to galactose-containing substrates and is normally found in the Golgi apparatus but can be proteolytically processed to a soluble form [62]	Differentially expressed in the adipose tissue of rat supplemented with genistein, a phytoeastrogen known to up-regulate the activity of the transcription factor PPARα [26] *ST6GALNAC4* is amongst one of the genes which was DE in response to the β 2-agonist, clenbuterol in pig adipose tissue and it was concluded that this gene may (along with other factors) contribute to adipose tissue reduction [63]
*LDLR*	lipid metabolic process - GO:0006629 cholesterol metabolic process - GO:0008203 regulation of triglyceride biosynthetic process -GO:0010867 phospholipid transport- GO:0015914 intestinal cholesterol absorption - GO:0030299 cholesterol transport- GO:0030301 low-density lipoprotein particle clearance - GO:0034383 lipoprotein metabolic process - GO:0042157 lipoprotein catabolic process - GO:0042159 cholesterol homeostasis - GO:0042632 cholesterol import- GO:0070508	The LDLR is a major determinant of plasma cholesterol levels. This cell surface receptor is expressed primarily in liver and removes cholesterol-carrying LDL from plasma by receptor-mediated endocytosis [64] The transcription of *LDLR* is primarily under the control of the transcription factor SREBP-2 [65] LDL-bound LDLR is endocytosed through a clathrin-dependent pathway and, after releasing in the late endosome, the LDLR is either recycled back to the plasma membrane or degraded in the lysosome [66] ADH is associated with mutations in the genes encoding *LDLR* and its ligand apolipoprotein B (APOB) [67]	Expression correlated with IMF % in pigs [68] Rare variants of *LDLR* have significant associations with familial hypercholesterolemia [69] PPARɣ activation has been shown to induce *LDLR* expression and enhance LDL cholesterol metabolism in a hepatic cell line [27] The transcription of LDLR is primarily under the control of SREBP-2 [65]
*MSMO1*	fatty acid metabolic process- GO:0006631 fatty acid biosynthetic process-GO:0006633 cholesterol biosynthetic process -GO:0006695	Sterol-C4-methyl oxidase-like protein contains a set of putative metal binding motifs with similarity to that seen in a family of membrane desaturases-hydroxylases. The protein is localized to the endoplasmic reticulum membrane and is believed to function in cholesterol biosynthesis [70]	

**Fig. 2:**
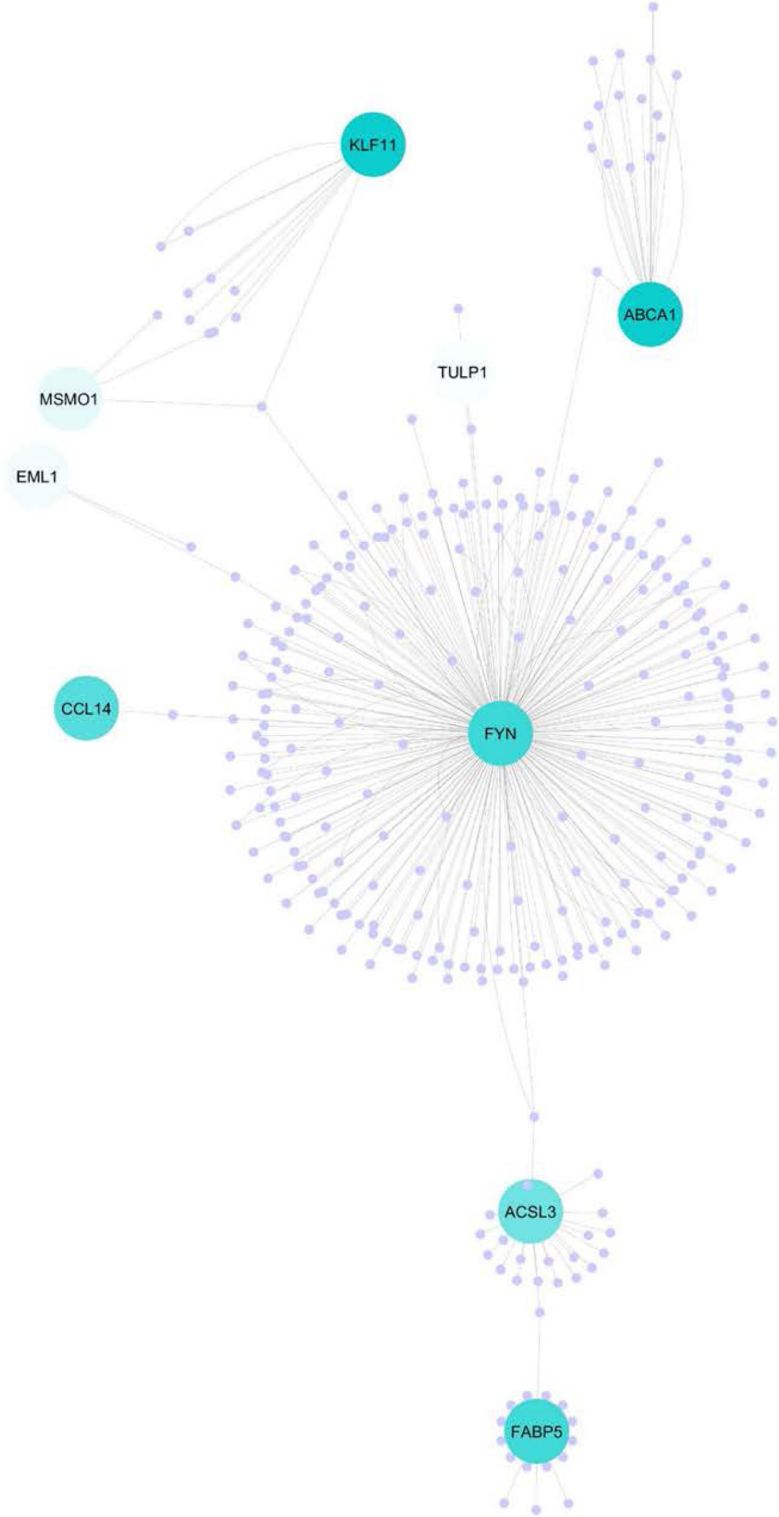
A topographical representation (Edge-Weighted Spring Embedded layout) of PPI network generated in Cytoscape™ for DE genes. The color intensity of the DE nodes are mapped to their fold change and unconnected genes are excluded

The top canonical pathways identified by IPA included: 1) Mitochondrial L-carnitine Shuttle Pathway (*P* = 2.06 × 10 ^−4^), 2) LPS/IL-1 Mediated Inhibition of RXR function (*P* = 2.62 × 10 ^−3^), 3) Acetate Conversion to Acetyl-CoA, 4) Tetrapyrrole Biosynthesis II (*P* = 7.58 × 10 ^−3^) and 5) Zymosterol Biosynthesis (*P* = 7.58 × 10 ^−3^). Network analysis and visualisation of protein-protein interactions (PPI) using Cytoscape™, highlighted *FYN* as an important hub gene connecting a number of the DE expressed genes (Fig. 2).

### Validation of microarray data and analysis

**Fig. 3:**
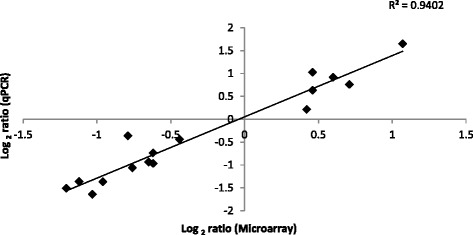
Scatter plot of mean log_2_ ratio of normalised relative quantities for QPCR v Microarray (*n* = 14)

**Fig. 4:**
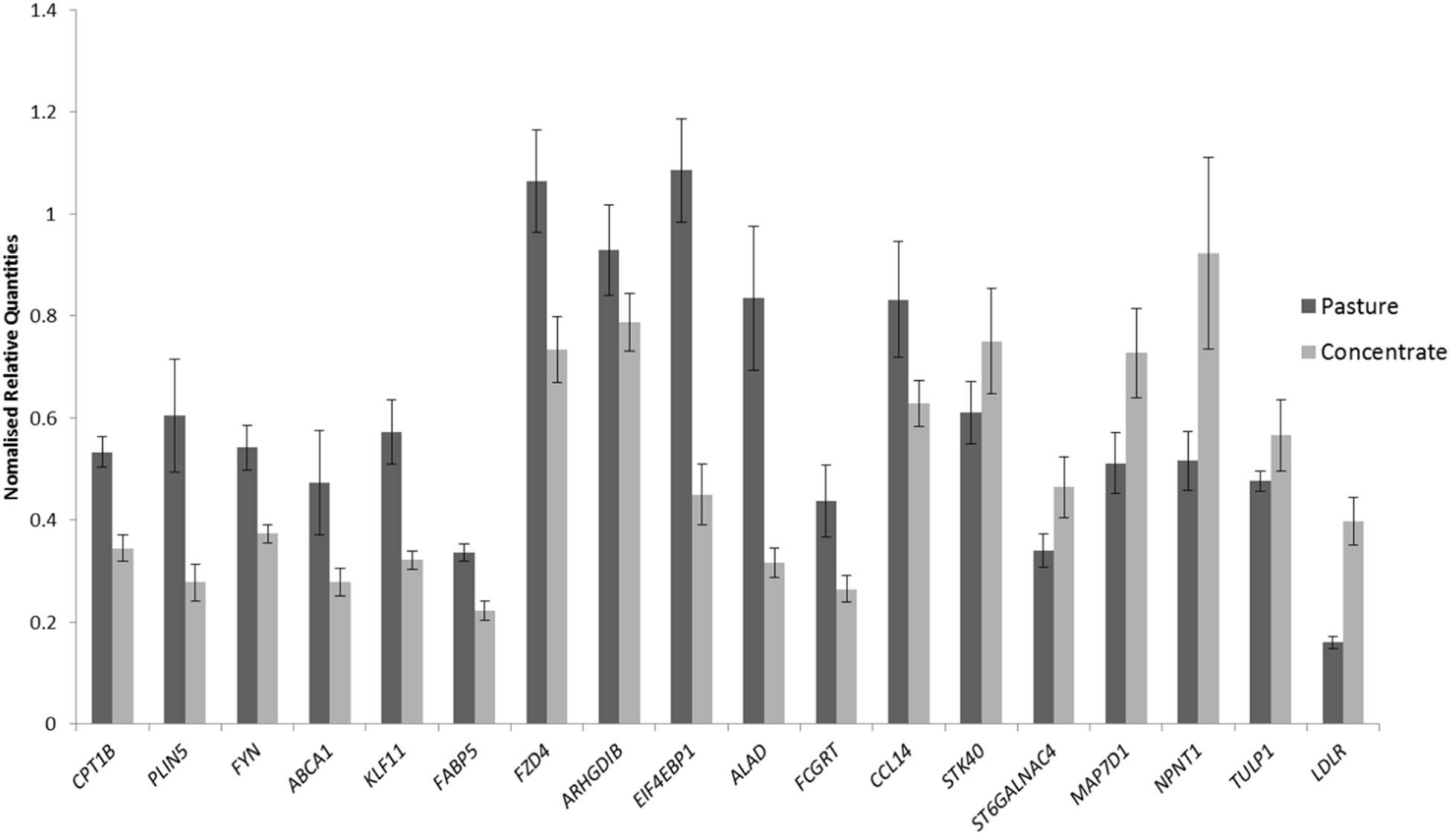
Plot of QPCR results in an independent cohort of pasture-fed (*n* = 16) and concentrate-fed (*n* = 16) animals on a selected subset of DE genes

**Fig. 5:**
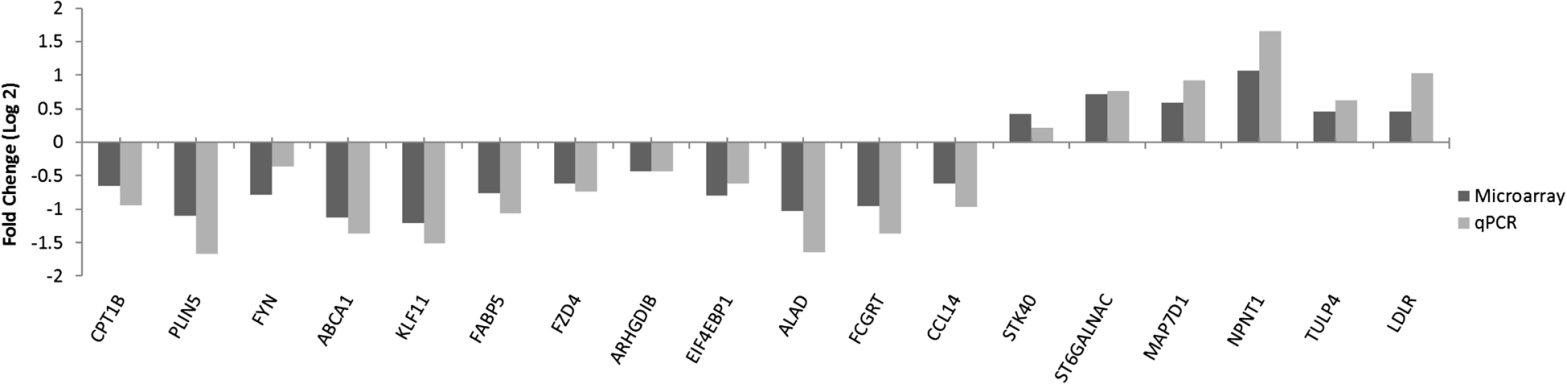
Fold change (Log_2_) for QPCR and Microarray (*n* = 14)

Quantitative PCR (QPCR) assays were carried out on a subset of the 26 genes that were identified as DE in the microarray analysis as a means of validating the microarray analysis, normalized relative quantities are presented in Additional file 2: Data set S1. A strong correlation (r^2^ = 0.94) was observed between the gene expression data generated from the microarray and QPCR assays (Fig. 3). In the larger cohort of animals (outdoor/pasture-fed (*n* = 22) and indoor/concentrate-fed (*n* = 22)), 15 of the 17 genes analysed were DE between the outdoor/pasture-fed and indoor/concentrate-fed groups (*P* < 0.05) (Fig. 4). *ARHGD1B* and *STK40* were not significantly different (*P* > 0.05) although the direction of regulation between the microarray and QPCR results was the same (Fig. 5).

### Associations with fatty acid profiles of the muscle and DE genes

**Fig. 6:**
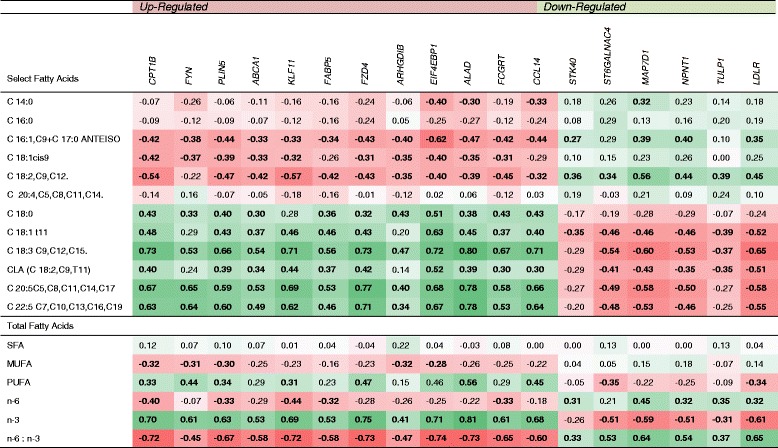
Heat map illustrating Pearson correlation (r^2^) of normalised relative expression of DE genes and selected fatty acids, total fatty acids and n-6:n-3 fatty acid ratio, for outdoor/pasture-fed and indoor/concentrate-fed animals (*n* = 44) Significant associations (*P* < 0.05) are highlighted in bold

The concentrations of a number of FA which formed the basis of a previous publication (Röhrle FT, Moloney A, Lejonklev J, Osorio MT, Monahan FJ: Discrimination of beef from different production systems and countries based on muscle fatty acids, Submitted) [71] were correlated with the expression levels of the up-regulated genes in the pasture-fed group. Most notably, the total n-3 FA concentration in the muscle was positively correlated with the majority of these up-regulated genes, while total n-6 FA concentration was negatively correlated with the up-regulated genes and positively correlated with the down-regulated genes (Fig. 6). This was also evident from the scatter plots between gene expression and n-3 FA concentration (Additional file 3: Figure S3). With the exception of conformation there were no differences between the carcass and IMF characteristics of animals offered grass or concentrate diets (Table 3).

**Table 3:** Carcass and IMF characteristics of outdoor/grass-fed and indoor/concentrate-fed animals

Trait	Outdoor/Grass-fed	Indoor/ Concentrate-fed	S.e.d.	Significance
Initial weight (kg)	275.5	273.0	2.49	NS
Final weight (kg)	512.7	506.1	7.63	NS
Carcass weight (kg)	270.2	276.8	4.23	NS
Fatness^a^	3.16	2.90	0.17	NS
Intra-muscular Fat (g/kg)	30.9	41.2	11.6	NS

^a^Conformation: Excellent = 5, Poor = 1; Fatness 1 = lean, 5 = fat (4L = 3.75)

### Principal component analysis

**Fig. 7:**
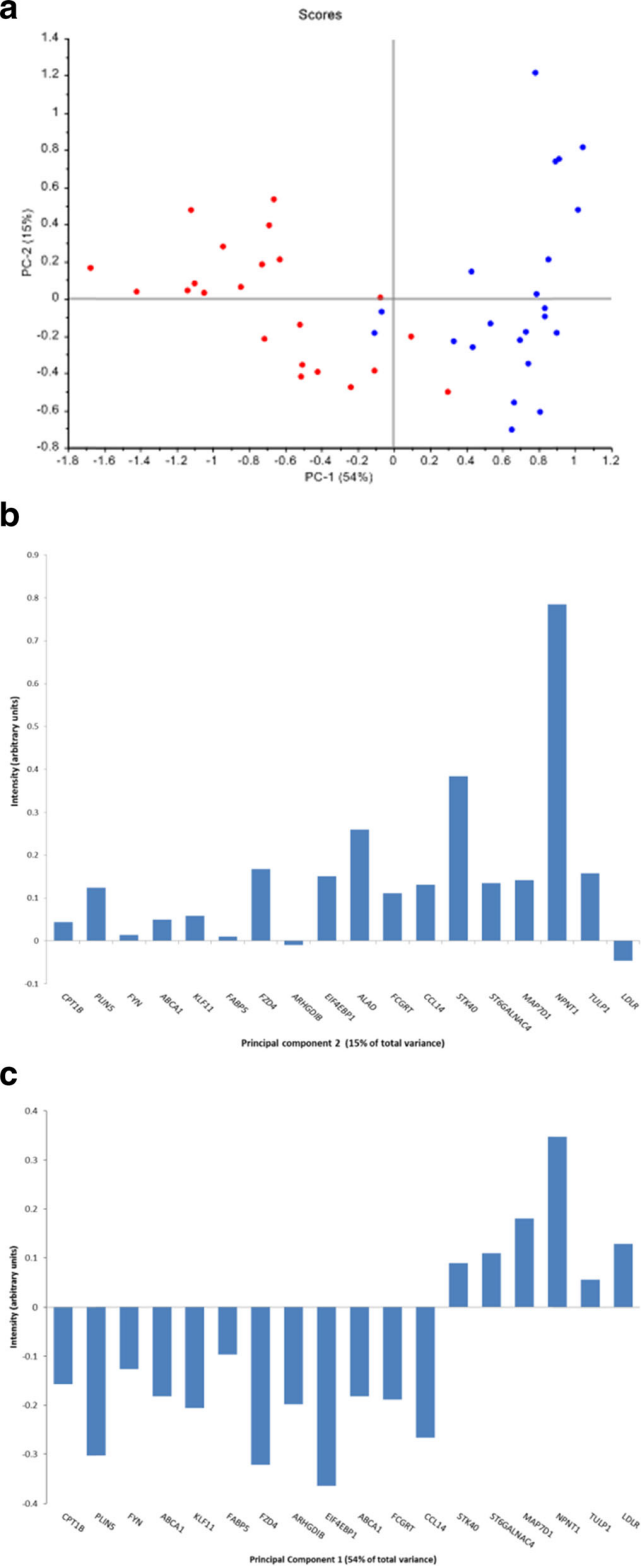
**a** Principal components 1 and 2, **b**) loading plot for principal component 1 and **c**) loading plot for principal component 2

Principal component analysis (PCA) was performed on the entire QPCR dataset of 44 beef samples to identify any existing clustering behaviour (no mathematical pre-treatment was applied to the gene data). Principal components (PC) 1 and 2 accounted for 54 and 15 %, respectively, of the variance in this dataset; a score plot on these two components revealed an almost complete separation of samples on the basis of the production system (Fig. 7a). Only two samples of each class i.e. outdoor/pasture-fed and indoor/concentrate-fed were mis-classified in the score plot. In general, the concentrate-fed animals exhibited a greater dispersion along PC1 than the outdoor/pasture-fed animals, a trend which was reversed on PC2. The loading plot for PC1 (Fig. 7b) can be used to indicate which variables were responsible for the sample clustering observed. Loading 1 reveals that the location of indoor/concentrate-fed animals was determined mainly by genes with positive values in the loading plot although it is the cumulative effect of all genes that determines a sample score in PC space. Similarly for PC2, the greater spread of concentrate-fed animals is particularly influenced by expression levels of *NPNT* and, to a lesser extent, *STK40* (Fig. 7c).

To determine the minimum number and identity of genes necessary to effect the separation of the two animal classes, genes were de-selected in a 5 step procedure (based on regression coefficient magnitudes), and the PCA analysis was re-run after each deletion step. The effect of these deletions was monitored by tracking correct classification rates for each PCA model; these were stable at four mis-classifications (2C + 2P, 2C + 2P, 2C + 2P, 1C + 3P and 1C + 3P, respectively (C = indoor/concentrate-fed, P = outdoor/pasture-fed) until, at the final deletion step, only three genes (*ALAD*, *EIF4EBP1* and *NPNT*) were retained in the analysis. Classification based on these genes resulted in a classification rate of 95 % of the outdoor/pasture-fed animals (misclassification of two outdoor/pasture-fed animals) and 100 % of indoor/concentrate-fed animals. Several other three-gene permutations were studied but none could model the data as well as the combination of *ALAD*, *EIF4EBP1* and *NPNT*.

### Discriminant analysis and support vector machine (SVM)

Discriminant analysis was applied to this dataset using discriminant partial least squares (D-PLS) regression and linear discriminant analysis. Models were developed on a calibration sample set which contained 50 % of each of the two sample groups i.e. 11 the outdoor/pasture-fed and 11 indoor/concentrate-fed. The model produced from this latter exercise was then used to predict the class affiliation of the remaining 50 % of the samples. All of these methods produced the same result i.e. 20 of the 22 prediction samples were correctly classified. The same 2 the outdoor/pasture-fed samples identified as clustering anomalously in the PCA score plot were mis-classified in each of these cases. Both models correctly classified 95.45 % of the animals i.e. 42 of 44 animals. Both of the mis-classified animals belonged to the outdoor/pasture-fed group. The most accurate SVM model produced correct classification rates of 100 % for the concentrate-fed animals and 95.45 % (1 mis-classification out of 22) for the the outdoor/pasture-fed animals.

### Regulatory elements within the promoter region of the differentially regulated genes

The pattern of co-expression of the DE genes suggests there may be a common regulator. The up-regulated genes were correlated with each other, the down regulated genes were correlated with each other, while both groups of genes were in negative correlation with each other. This trend is illustrated clearly in a heat map (Additional file 4: Figure S4). To explore this possibility the DE genes were analysed using a range of *in-silico* approaches with the aim of uncovering common regulators that may influence their expression. A number of upstream regulators were identified in Ingenuity Pathway Analysis, including ATP7B (*P* = 1.02 ×10 ^−5^), meldonium (*P* = 3.2 × 10 ^−5^), cholesterol (*P* = 3.96 × 10 ^−5^) and SREBF2 (*P* = 5.98 × 10 ^−5^). *In-silico* analysis of the promoter sequences of the bovine DE genes, supported the hypothesis that these putative transcription factor binding sites (TFBS) are present in the bovine promoters.

A number of histone modifications were significantly over-represented in the region 1kb upstream of the human orthologues when analysed using a web resource (cSCAN) which collates data from genome-wide ChIP-Seq experiments performed on transcription factors and histone modifications (Additional file 5: Figure S5) [72]. Selected histone modifications derived from cSCAN present within the muscle cell line (HSMM), along with selected putative regulatory elements identified within the bovine promoters (1 Kb upstream of start site) are summarised in (Additional file 5: Table S1).

## Discussion

The identification of a panel of DE genes in the muscle of cattle raised outdoors on pasture compared to cattle raised indoors on concentrate feed offers the potential for transcriptomic data to be used in discriminant approaches to classify beef based on its production system. The finding that the relative expression data from three genes; *ALAD*, *EIF4EBP1* and *NPNT* is sufficient to model the two production systems in this study is a promising step with regard to authentication. The transcriptomic data generated from this experiment enabled the correct classification of 95 % of outdoor/pasture-fed animals and 100 % of indoor/concentrate-fed animals.

Of significance in this study was the fact that a substantial proportion of the DE genes were unequivocally involved in FA metabolism in particular β-oxidation and FA transport. In this regard, the findings are very consistent with alterations in enzyme activities observed in cattle fed on perennial ryegrass pasture versus those fed on a more concentrated maize silage ration in a similar authentication study performed by Cassar-Malek et al*.* in 2009 [73]. Here, the authors reported an ‘oxidative switch’ in response to pasture diet with no effect on the muscle’s glycolytic metabolism. At a transcriptomic level however, the DE genes identified in this study contrast to those highlighted by Cassar-Malek et al*.* in 2009, where Selenoprotein W was highlighted as the most promising classifier and the three main categories of DE genes related to oxidative phosphorylation, contraction and protein metabolism not lipid metabolism [73].

More surprisingly, this present study is highly consistent at the transcriptomic level with an *in vitro* study which examined PPARδ activation in human myotubes [21]. In this study, five of 21 differentially upregulated genes (in response to PPAR activation) were also upregulated in the muscle of the outdoor/pasture-fed animals, including; *CPT1A, KLF11, PLIN2* (*PLIN5* in this study), *ABCA1* and *ACSL3*. It was concluded from the study that the principal effect of PPAR activation in human myotubes was an increased mitochondrial fatty acid oxidative capacity, but not mitochondrial biogenesis [21]. The peroxisome proliferator-activated receptor γ coactivator-1 (PGC-1) family of transcriptional coactivators are central in transducing and integrating physiological signals to the transcriptional machinery controlling mitochondrial functional capacity [74]. Many of the genes reported to be DE are downstream targets of PPAR or key to the regulation of mitochondrial fatty acid oxidation e.g. Carnitine palmitoyltransferase 1 [75].

A number of the up-regulated genes in the outdoor/pasture-fed animals are fundamental to the β-oxidation of fatty acids in muscle tissue, including *CPT1B, PLIN5*, *FYN* and *FABP5.* Carnitine palmitoyltransferase 1B (*CPT1B*) is involved in the transport of long chain fatty acids through the mitochondrial membrane and is the rate limiting step in lipid catabolism [76]. Interestingly, a separate study reported that *CPT1B* was up-regulated in the ST muscle of grazing lambs relative to indoor lambs [30]. *PLIN5* displays an ability to both regulate oxidative gene expression and to facilitate the release of FA from muscle for mitochondrial oxidation and is specifically expressed in cells that actively oxidize FA such as red muscle [33]. Fatty acid binding protein 5 (*FABP5)* promotes the transport of FA in the cytoplasm to organelles such as the endoplasmic reticulum for triacylglycerol and cholesterol synthesis and for lipoprotein assembly [44, 77] and shuttles ligands from the cytosol to the nuclear receptor PPARβ/δ thereby enhancing the transcriptional activity of the receptor [45]. An exploration of the topographical arrangement of the DE expressed genes in Cytoscape™ [78] highlighted *FYN* as a hub gene with extensive connectivity to several genes including a large number of the DE genes, i.e. up-regulated; *CCL14, KLF11, ABCA1, FABP5, ACSL3* and down-regulated; *MSMO1, TULP1, EML1* (Fig. 2). FYN is a tyrosine specific phospho-transferase and a member of the large Src family of non-receptor tyrosine kinases, which forms a ternary protein complex composed of FYN, LKB1, and the master metabolic regulator/sensor, adenosine monophosphate-activated protein kinase (AMPK) is assembled with CD36 [79]. When exogenous FA concentrations are low, CD36-bound FYN can access and phosphorylate LKB1, which induces LKB1 nuclear relocation, thus reducing the cytosolic LKB1 concentrations available to activate AMPK. Hence, when exogenous FA concentrations are low, AMPK is quiescent. However, when exogenous FA concentrations rise, FYN dissociates from the protein complex, allowing the cytosolic LKB1 to activate AMPK, thus enhancing FA oxidation through the inactivation of acetyl-CoA carboxylase [80].

The profile of DE genes are supported by the FA analysis on the muscle of animals raised outdoors on pasture, where an increase in the proportions of total n-3 PUFA, specifically (C18:3n-3; C20:5n-3; C22:5n-3 and C22:6n-3) was observed, relative to those raised indoors on concentrates [71]. In addition, the majority of the up-regulated genes correlated positively with total n-3 PUFA and negatively with n-6 PUFA concentrations in the muscle of these animals, indicating that these genes could be altering the n-3/n-6 PUFA ratio in a coordinated manner within the skeletal muscle of outdoor/pasture-fed cattle. These findings concord with the wider literature, with respect to the measurable changes observed in the FA profiles of meat derived from the outdoor/pasture-fed animals [81–83].

The relationship between rearing animals outdoors on pasture and the deposition of certain FA in muscle and adipose tissue is well established [82–84]. Thus, C18:3n-3, C22:5n-3 and C22:6n-3, are typically elevated in pasture-fed animals compared to animals fed cereal concentrates. The higher levels of these FA in beef from pasture-fed animals can be attributed directly to the higher C18:3n-3 content of the grass (approximately 49 % of total FA) compared to cereal concentrates (approximately 2 % of total FA) [82]. The reverse is true of the n-6 FA, C18:2n-6 and its long chain counter parts, such as C20:4n-6, which are higher in beef from concentrate-fed animals and reflect the higher C18:2n-6 content of cereal concentrates relative to pasture [82]. Interestingly, no relationships were found between the DE genes and conjugated linoleic acid, which is typically elevated in beef from pasture-fed animals and also in the outdoor-pasture reared animals in this study [82, 83].

The differential expression of the genes highlighted in this study may be attributed to a number of variables including: 1) greater availability of PUFA, particularly n-3 PUFA in the diet (pasture-fed), 2) differences in the foraging behavior between the outdoor/pasture and indoor/concentrate-fed animals and 3) greater availability of bioactive bioflavonoids in the pasture. In relation to the first point, a number of genes have been reported to be regulated by FA, including genes involved in FA transport, activation of FA, mitochondrial β-oxidation and peroxisomal oxidation. It has been reported that the regulation of gene transcription by FA is due to changes in the activity or abundance of at least four different transcription factor families including PPAR, liver x receptor (LXR), hepatic nuclear factor 4 (HNF-4) and sterol regulatory element binding protein (SREBP) [85]. Analysis of the transcriptome of porcine muscle indicated that animals with higher percentages of PUFA exhibit a shift toward a more oxidative metabolic state and exhibit increased mitochondrial function, FA uptake and oxidation [86]. This supports the concept that a greater availability of n-3 PUFA in the diet is directly influencing the muscle transcriptome especially with respect to its FA metabolic functioning.

Also relevant to this study is the fact that the outdoor/pasture-fed animals are afforded the opportunity to exercise their natural behavioral pattern [46] which for cattle reared on pasture includes significant periods of foraging or food-search behaviour [87, 88]. This greater exposure to exercise (in the form of foraging) is another potential environmental contributor to the differential gene expression observed in the outdoor/pasture-fed group [89]. It is widely established that endurance exercise promotes phenotypic adaptations in skeletal muscle causing a shift toward a more oxidative phenotype. Endurance type exercise favours the growth and expression of type I and type IIa (type IIx) muscle fibres [90]. Oxidative fibres contain a high density of mitochondria and preferentially utilise FA as a source of energy while fast contracting glycolytic fibres which contain fewer mitochondria use relatively more glucose in this context a relationship between exercise (which is likely to be higher in the outdoor/pasture-fed group) and increased proportions of oxidative type I muscle fibres has been established [91, 92].

While gene regulation was not examined experimentally in this study, some of the *in-silico* analysis suggests that there may be common regulatory influences underpinning the correlated expression of the DE genes in this study. IPA identified a number of upstream regulators relating to FA metabolism including cholesterol and SREBF2. Putative transcription factor binding sites relating to adipogenesis were also identified in regions upstream of the DE expressed genes including PPAR, CCATT/enhancer binding protein (C/EBP), SCREB and GATA. Literature searches on all the DE genes identified other regulators such as microRNAs which were relevant to *LDLR* and *ABCA1*. The most dominant regulator of these DE genes which was cited in the literature, highlighted in the pathway analysis and inferred from other experiments was PPAR. PPARs constitute a family of ligand-dependent nuclear receptors that are activated by all long-chain FA or their derivatives and also specific synthetic ligands [29]. In addition to this PPARδ expression is increased by exercise training in both rodents and humans, but in addition to this, AMPK and PPARδ agonists are recognised as exercise mimetics [93]. Hence, the greater availability of PPAR ligands in the form of PUFA from the diet combined with increased endurance type exercise might together drive the formation of a more oxidative phenotype in the muscle thereby up-regulating PPAR responsive genes. The analysis of protein-protein interaction (PPI) networks in the context of the DE genes, highlighted FYN as an important hub gene connecting a number of the DE genes. Both FYN and AMPK have been shown to be implicit in linking FA uptake to β-oxidation in myocytes [80]. In addition, many small molecules and phytochemicals derived from plants have similar chemical structures to known kinase inhibitors that can inhibit FYN and other Src family kinases [94, 95]. Finally, the *in silico* analysis highlighted some histone modifications as being significantly over-represented in human paralogues of the down-regulated gene list. This epigenetic angle is also is supported by a recent study which identified global reductions in acetylation of histones and increased methylation of specific genes in DNA derived from the mammary tissues of animals receiving a high-concentrate diet versus a mixed forage diet [95].

While this study highlights *ALAD, EIF4EBP1* and *NPNT* as potential transcriptomic classifiers of the two contrasting production systems, it also highlights potential regulators and drivers which may be influencing the pattern of expression of the DE genes with subsequent effects of the n3/n6 FA ratio within the muscle. However, these potential classifiers would require validation across a wider range of breeds, which naturally vary in their n-3/n-6 ratios irrespective of production system [96]. Also different geographical contexts and other permutations implicit in the production systems which effect both diet and exercise could also impact on the transcriptome in an as yet unforeseen way. If however, the genes highlighted in this study are powerful enough to discriminate across a wider range of contexts, there may be the potential to develop this information into a robust assay e.g. an ELISA system poses less technical barriers, is less time consuming and more robust than using relative quantities derived from gene expression data.

## Conclusion

In this study, the outdoor/pasture-fed group were characterised by the differential expression of 26 genes in muscle tissue, compared to the indoor/concentrate-fed group. A significant number of these genes are involved in FA metabolic processes including β-oxidation; PPAR and AMPK/FYN were highlighted as potential regulators. The differences in the dietary availabilities of PUFA and bioflavonoids as well as alterations to the muscle physiology due to differential foraging habits between the two production systems are proposed drivers of the observed differential expression. The normalised expression levels of three genes *ALAD, EIF4EBP1* and *NPNT* are sufficient to model the two production systems types in this context and hence this data has potential in the wider setting to be developed as a means of authenticating production systems. The robustness of the DE genes would need to be tested across a broader range of contexts to further determine their potential as classifiers, as different breeds and muscle types have contrasting characteristics with respect to both oxidative capacity and FA deposition, irrespective of production system.

## Methods

The trial was conducted under experimental license from the Irish Department of Health and Children in accordance with the Cruelty to Animals Act 1876 and the European Communities (Amendments of the Cruelty to Animals Act, 1976) Regulations, 1994 and with the approval of Teagasc, the Irish Agricultural and Food Development Authority. The cattle used in this study were purchased by Teagasc.

### Animals and feed regimen

The animals reported here were part of a larger study described by Röhrle et al*.* [12]. Charolais × Limousin crossbred heifers (mean age 252 ± 28 (s.d.), mean weight 275 ± 27 kg) were randomly assigned to treatment for 12 months prior to slaughter: a pasture-fed group and a concentrate-fed group. The outdoor/pasture-fed group (*n* = 25) grazed outdoors on a pasture consisting mainly of *Lolium perenne L., Poa spp. and Trifolium repens L.* The daily target herbage dry matter (DM) intake of 0.02 of live weight per heifer above a residual or post-grazing herbage mass of 900 kg DM ha^−1^. The daily allowance was provided by adjusting the grazing area based on an estimate of grass DM yield/ha. The indoor/concentrate-fed group (*n* = 25) were permanently housed and received concentrate and barley straw. The indoor/concentrate group were managed in 5 groups of 5 animals per group. The outdoor/pasture-fed group were managed as 3 groups, consisting of 8, 8 and 9 per group. The composition of the concentrate was 430 g kg^−1^ pelleted beet pulp, 430 g kg^−1^ rolled barley, 80 g kg^−1^ soybean meal, 35 g kg^−1^ molasses, 20 g kg^−1^ mineral/vitamin premix and 5 g kg^−1^ lime. The concentrate was offered once daily at an allowance calculated to ensure a similar growth rate to the heifers at pasture and straw was offered at approximately 25 % of dietary DM. Over the duration of the study the mean daily DM intake of the concentrate-fed group was 4.62 kg concentrate and 1.56 kg straw.

### Sample collection

Animals were slaughtered in accordance with European regulations at Meadow Meats Limited, Rathdowney, Co Laois. This study was carried out under licence from the Irish Government Department of Health and Children and with the approval of Teagasc, the Agricultural and Food Development Authority. All procedures used complied with national regulations concerning experimentation on farm animals. Samples from the *M. longissimus dorsi* (LD) muscle were collected within 20 min postmortem. Approximately 50g of muscle sample was taken from above the 11th and 12th rib. The muscle was dissected aseptically into smaller pieces and was stored in RNALater™, (Ambion Ltd., Cambridge, UK) for 24 h and subsequently the RNALater™ was removed and the sample was transferred to −80 °C for long term storage.

### Total RNA extraction

Total RNA was extracted using Trizol® reagent (Sigma-Aldrich Corp., St. Louis, MO, USA) and TissueLyzer ™ (Qiagen, Hilden, Germany) according to the manufacturer’s protocols. The extracted RNA was treated with DNase I (Qiagen, Hilden, Germany) at room temperature for 10 min to remove genomic DNA. Total RNA was quantified and assessed for purity on a NanoDrop™ 1000 Spectrophotometer (Thermo Fisher Scientific, Waltham, MA, USA) and samples with a 260/280 ratio ≥ 2.0 were considered suitable for cDNA synthesis. Total RNA integrity number (RIN) was assessed on the Agilent 2100 Bioanalyser version A.02.12 (Agilent Technologies Inc., CA, USA) using an RNA 6000 Nano LabChip kit (Caliper Technologies Corp. MA, USA).

### Amplification of total RNA

All samples were used for QPCR analysis, with seven samples randomly selected per treatment group for microarray analysis. Total RNA (50 ng) from each sample was amplified using the NuGEN WT-Ovation FFPE RNA Amplification System (NuGEN Technologies, San Carlos, CA) in accordance with the manufacturer’s instructions. First-strand synthesis of cDNA was carried out using a unique first-strand DNA/RNA chimeric primer mix, resulting in cDNA/mRNA hybrid molecules. Following fragmentation of the mRNA component of the cDNA/mRNA molecules, second-strand synthesis was carried out and double-stranded cDNA was formed with a unique DNA/RNA heteroduplex at one end. In the final amplification step, RNA within the heteroduplex was degraded using RNaseH, and replication of the resultant single-stranded cDNA was achieved through DNA/RNA chimeric primer binding and DNA polymerase enzymatic activity. The amplified single-stranded cDNA was purified using the Zymo Research Clean & Concentrator-25 kit (Zymo Research, Irvine, CA) to allow for accurate quantitation of the cDNA and to ensure optimal performance during the fragmentation and labeling process. The single-stranded cDNA was assessed for quantity and quality using the NanoDrop™ 1000 Spectrophotometer (Thermo Fisher Scientific, Waltham, MA, USA) in combination with the Agilent Bioanalyzer™ (Agilent Technologies Inc., CA, USA).

### Fragmentation and labeling of amplified single-stranded cDNA

Five μg of the amplified single-stranded cDNA was fragmented and labeled using the FL-Ovation cDNA Biotin Module V2 (NuGEN Technologies, San Carlos, CA) in accordance with manufacturer’s guidelines. The enzymatically and chemically fragmented products (50–100nt) were labeled via the attachment of biotinylated nucleotides onto the 3′ end of the fragmented cDNA.

### Hybridisation onto Affymetrix GeneChip arrays

One Affymetrix® GeneChip Bovine Genome Array (Affymetrix UK Ltd., high Wycombe, UK) was used per animal (pasture-fed (*n* = 7) and concentrate-fed (*n* = 7)). The fragmented and labeled cDNA was added to the hybridisation cocktail in accordance with the NuGEN guidelines (NuGEN Technologies, San Carlos, CA). Following hybridisation for 18 h at 45 °C, the array was washed and stained on the GeneChip Fluidics Station 450 (Affymetrix, Santa Clara, CA), inserted into the Affymetrix autoloader carousel and scanned using the GeneChip Scanner 3000 (Affymetrix, Santa Clara, CA). Quality control information relating to the arrays is available in (Additional file 6: Dataset S2 and Additional file 7: Figure S6-S11).

### Microarray quality and data analysis

The microarrays were processed using a custom pipeline written in R using Bioconductor libraries. The Bioconductor packages “affy” and “affyPLM” [97] were used to generate images, histograms, box plots, degradation plots, MA plots and scatter plots to evaluate the quality of the hybridized arrays. The arrays were read in using functions from simpleaffy [98]. The data were pre-processed and normalised using the Factor Analysis for Robust Microarray Summarisation (FARMS) algorithm [99]. The data were normalised using the quantile normalisation technique as implemented in the qFarms function. The normalised probeset data were then filtered to retain only the informative probes as determined by FARMS. The filtered expression set was then analysed to identify DE genes using the empirical bayes (eBayes) function in Linear Models of Micro Array data (LIMMA) package [100] contained within the R statistical package. Genes displaying differential expression were then annotated using the Affymetrix® bovine gene annotation. The design matrix was generated using the puma library [101]. A Benjamini-Hochberg false discovery rate of 0.05 was used as the cut-off for significance [102]. Significant probes were annotated using the bioconductor AnnotationDbi [103] and bovine.db libraries [104]. The expression data generated for the current study are MIAME-compliant [105] and are deposited in the NCBI Gene Expression Omnibus (GEO) repository [106] with experiment series accession GSE52145.

### Functional classification of differentially expressed genes

To understand biological meaning and perform functional classification of DE genes two exploration tools were used: IPA, version 8.7 (Ingenuity Systems, CA, USA), and Database for Annotation, Visualization and Integrated Discovery (DAVID) [107]. IPA and DAVID are both web-based software applications that allow identification of pathways, biological network and functions of experimental data and gene lists. Cytoscape [78] was used to visualise the topographical arrangement of DE genes based on their binary interactions which were imported from the IntAct PPI database [108]. Statistical analysis of the network was performed using the NetworkAnalyzer plugin of Cytoscape [109].

### Microarray validation

Quantitative PCR (QPCR) assays were designed and validated for 17 (11 up-regulated and 6 down-regulated in the pasture-fed group) of the 26 genes that were identified as DE in the microarray analysis. These genes were selected based on a cut off of between +0.4 and −0.5 (Log fold change) from the microarray analysis. The relative expression of these genes was assessed in a larger set of outdoor/pasture-fed (*n* = 22) and indoor/concentrate-fed (*n* = 22) animals (Additional file 2: Data set S1). Primers were designed using Primer Express™ 3.0 software (Applied Biosystems, Warrington, UK) (Additional file 8: Table S3). The specificity of the QPCR assay was assessed using dissociation curve analysis and only assays with efficiencies between 90-110 % were used. Primers for the reference ribosomal protein large (*RPLP0*) and Tyrosine 3-Monooxygenase (*YWHAZ*) were as previously described [110]. These assays were performed on the muscle tissues collected from the pasture (*n* = 22) and concentrate (*n* = 22) maintained animals (the 7 animals that were analysed in the microarray experiment were included in these cohorts).

Random hexamer primed cDNA synthesis was performed using 1 μg of total RNA and SuperScript III Reverse Transcriptase (Invitrogen, Carlsbad, CA) in a final volume of 20 μL according to manufacturer’s recommendations.

Each QPCR reaction was carried out in duplicate in a 10 μL reaction mixture containing 1 μL cDNA, 5 μL Power SyBr Green PCR master mix (Applied Biosystems, Warrington, UK) and primers at a final concentration of 300 nM. QPCR was carried out on an ABI 7300 real-time PCR system (Applied Biosystems, Warrington, UK) using the following conditions: 50 °C for 2 min, 95 °C for 10 min, 40 cycles at 95 °C for 15 s and 60 °C for 1 min.

The raw Ct values for the reference genes were converted to relative quantities using the formula Q = E ^Δ^Ct where E is the PCR efficiency of the assay and ^Δ^Ct is the value calculated for the difference between the lowest Ct value and the Ct value of the sample in question for each gene. The relative quantities of the endogenous controls were then analysed for stability in geNorm [111]. The stability ‘M’ value generated by the geNorm application for the selected endogenous controls (*RPLP0* and *YWHAZ*) which was less than 1.5 indicated their suitability as endogenous controls for these muscle samples. The geometric mean of the relative quantities (normalisation factor) for *RPLP0* and *YWHAZ* was then calculated using geNorm. The relative quantities for the target genes were then divided by the normalisation factor for each sample to give the final normalised relative expression.

Correlation analysis was performed, between the DE genes and fatty acid data which formed the basis of a previous publication [71]. All samples had associated phenotypic data relating to the FA profiles of the muscle.

### Multivariate data analysis

All multivariate data analysis operations were performed using The Unscrambler X (version 10.3; Camo software, Oslo, Norway). Gene expression data were analysed without any transformation or other mathematical pre-treatment. Models were developed on a calibration sample set containing 50 % of samples while model validation used the remaining 50 %. Samples were selected for inclusion in the calibration set on a quasi-random basis i.e. every second sample in the data file was used with the remainder being allocated to the validation set. Models were developed using full i.e. leave-one-out cross-validation. Principal component analysis is an unsupervised classification technique used for data visualisation and detection of unusual or outlying samples [112] in this case, the NIPALS algorithm was used after mean centering. Optimum models were identified on the basis of the first local minimum in the model residual variance plot in The Unscrambler. Discriminant PLS analysis [112] is a supervised discriminant procedure which requires the allocation of a dummy Y variable to each sample according to the class of animal involved. In this work samples of the outdoor class were assigned a Y value equal to 1 with indoor animals assigned a corresponding value of 0. In validation, samples with a predicted Y value ≥0.5 were assigned to category 1 while those with a predicted value <0.5 were identified as belonging to category 0. Support vector machine classification (SVM); [113] is a supervised machine learning technique which can handle some non-linearity in datasets. Important parameters in the application of this algorithm include the SVM type and kernel function; in this work, these were nu-SVC (nu value set to 0.5) and a radial basis function respectively. In all cases, classification model performance was assessed on the basis of correct classification rate (Röhrle FT, Moloney A, Lejonklev J, Osorio MT, Monahan FJ: Discrimination of beef from different production systems and countries based on muscle fatty acids, Submitted).

### In-silico identification of common regulatory components

The *In-silico* presence of regulatory motifs in the 5′ upstream region of the promoters of the DE genes were explored including: 1) CpG islands and putative TFBS present in the bovine promoters; and 2) over/under represented regulatory elements (including TFBS and histone modifications) based on data obtained from human ChIP-seq experiments.

CpG islands were identified from the Bovine Genome Browser [114] Baylor Btau_4.6.1/bosTau7 assembly, [Genbank: GCA_000003205.4]. A 1kb upstream region was downloaded for all of the DE genes from the Bovine UMD 3.1 assembly using the Ensembl genome browser [115] [Genbank: GCA_000003055.3]. This region was then analysed for the presence of putative TFBS relating to lipid metabolism using the JASPAR database [116].

As regulatory information is poorly annotated for genes in the bovine genome, a number of *in-silico* approaches using human paralogues were employed to gain an insight into any underlying regulatory influences that may be biologically relevant. Human paralogues of the DE genes were interrogated for the presence of regulatory elements (including TFBS and histone marks) within a skeletal muscle cell line (HSMM) reported in the Cscan database [72]. The Human Ensembl transcript IDs corresponding to the DE genes were obtained using DAVID (Additional file 5: Table S2).

## Additional files

Additional file 1: Figure S1. Peroxisome proliferator-activated receptor (PPAR) pathway. **Figure S2.** Fatty acid degradation pathways (DOCX 332 kb) Additional file 2: Data set S1. Normalised relative expression of selected DE genes based generated from QPCR and primer pairs for DE genes. (XLSX 22 kb) Additional file 3: Figure S3. Scatter plots of Omega 3 fatty acid concentrations (units) versus normalised relative quantities for DE genes for Outdoor/pasture-fed (*n* = 22) and indoor/concentrate-fed animals (*n* = 22). (DOCX 177 kb) Additional file 4: Figure S4. Heat map and R^2^ values for selected DE genes derived from correlation matrix of QPCR data (*n* = 44). (DOCX 21 kb) Additional file 5: Figure S5. Outputs from cScan analysis for human transcripts a) up-regulated and b) down-regulated in the outdoor/pasture-fed group. **Table S1.** Summary of histone modifications identified on human paralogues and regulatory motifs on the upstream region of the bovine sequence of DE genes. **Table S2.** Human Ensembl IDs related to gene names of DE genes (a) up-regulated and (b) down-regulated in the pasture-fed group. (DOCX 210 kb) Additional file 6: Dataset S2. GeneChip quality control. (XLSX 25 kb) Additional file 7: Figure S6. Boxplot of unnormalised log intensity values. **Figure S7.** Boxplot of normalised log intensity values. **Figure S8.** MA-plots for un-normalised data. **Figure S9.** MA-plots for normalised data. **Figure S10.** RNA degradation analysis. **Figure S11.** Histogram of PM intensities for all 14 arrays. (DOCX 532 kb) Additional file 8: Table S3. Primer pairs used for QPCR on DE genes. (DOCX 16 kb)

## Abbreviations

C/EBP: CCATT/enhancer binding protein
DAVID: database for annotation, visualization and integrated discovery
DE: differentially expressed
D-PLS: discriminant partial least squares
FA: fatty acid
FARMS: factor analysis for robust microarray summarisation
GEO: gene expression omnibus
GO: gene ontology
IMF: intramuscular fat
IPA: ingenuity pathway analysis
LD: M. longissimus dorsi
LIMMA: linear models of micro array data
PCA: principal component analysis
PPAR: peroxisome proliferator-activated receptor
PPI: polyunsaturated fatty acids
PUFA: protein protein interaction
QPCR: quantitative PCR
SIMCA: soft independent modelling by class analogy
SVM: support vector machine
TFBS: transcription factor binding sites
