# Supplementary material for: The application of transcriptomic data in the authentication of beef derived from contrasting production systems
Source: BMC Genomics. 2016 Sep 21;17:746. doi: 10.1186/s12864-016-2851-7 (PMC5031250; doi:10.1186/s12864-016-2851-7)
Supplement: Additional file 1: Figure S1. — Peroxisome proliferator-activated receptor (PPAR) pathway. Figure S2. Fatty acid degradation pathways (DOCX 332 kb) [file 12864_2016_2851_MOESM1_ESM.docx]

Additional file 1


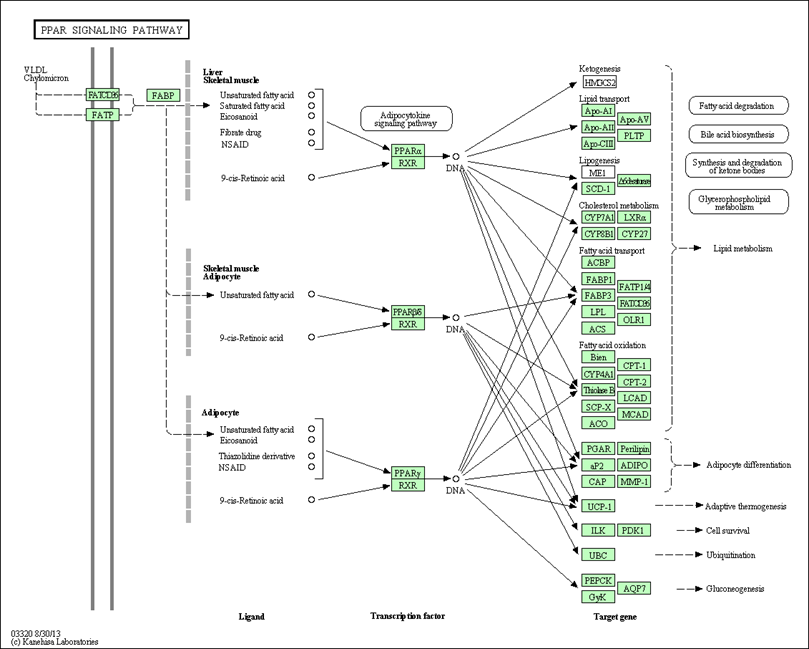


Fig S1 Peroxisome proliferator-activated receptor (PPAR) pathway


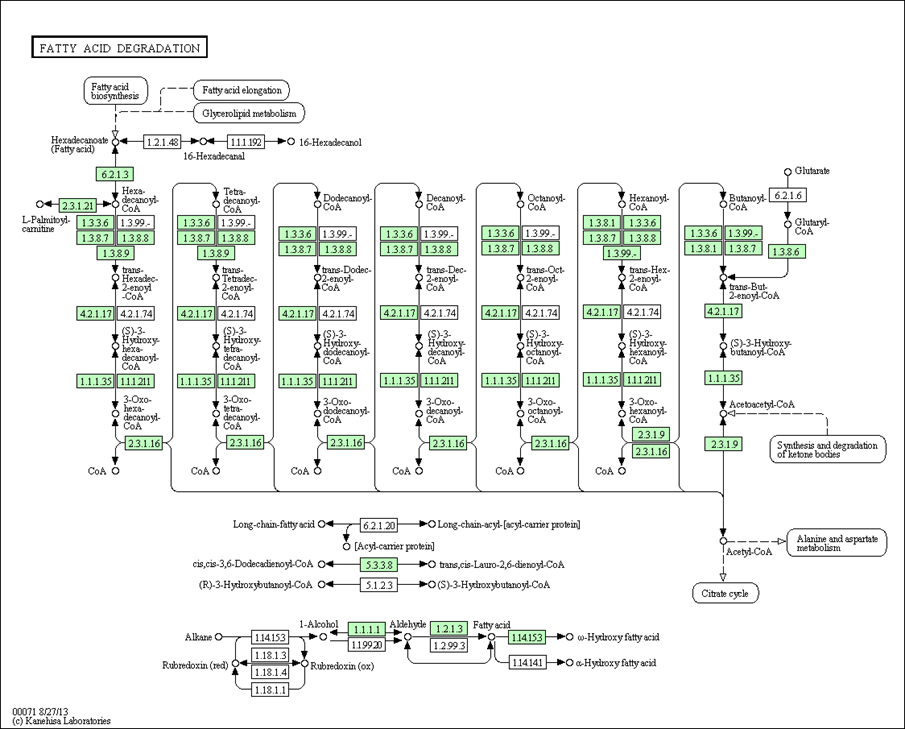


Fig S2 fatty acid degradation pathways
