# Supplementary material for: The application of transcriptomic data in the authentication of beef derived from contrasting production systems
Source: BMC Genomics. 2016 Sep 21;17:746. doi: 10.1186/s12864-016-2851-7 (PMC5031250; doi:10.1186/s12864-016-2851-7)
Supplement: Additional file 3: Figure S3. — Scatter plots of Omega 3 fatty acid concentrations (units) versus normalised relative quantities for DE genes for Outdoor/pasture-fed (n = 22) and indoor/concentrate-fed animals (n = 22). (DOCX 177 kb) [file 12864_2016_2851_MOESM3_ESM.docx]

| 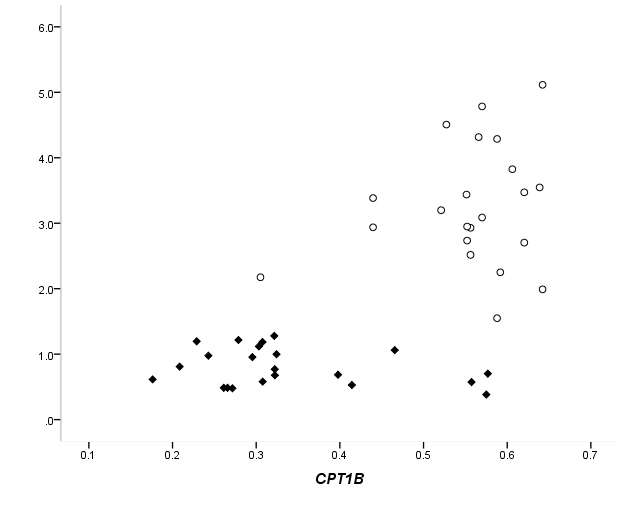 | 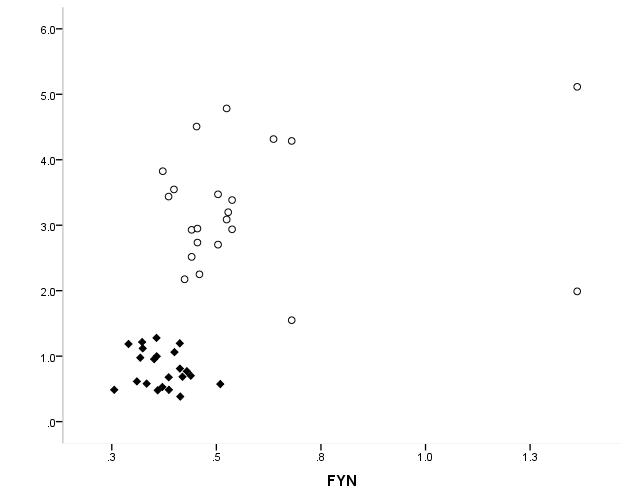 | 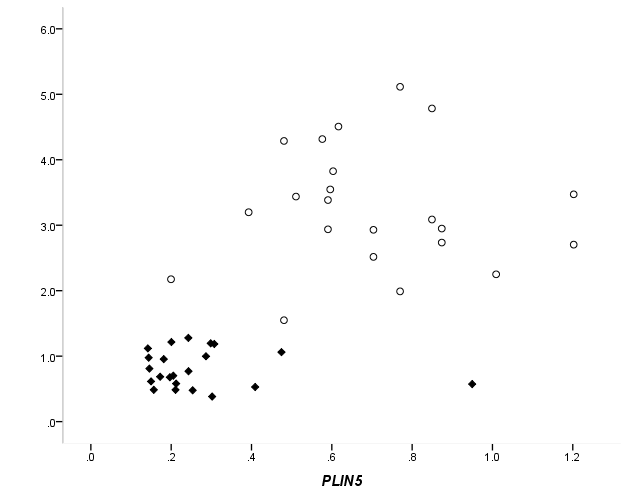 |
| --- | --- | --- |
| 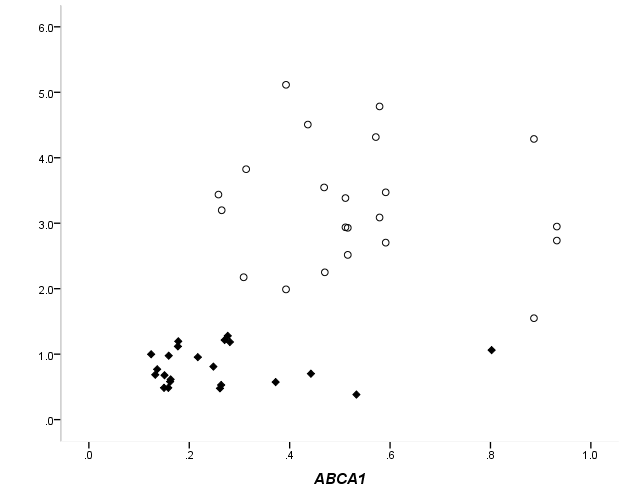 | 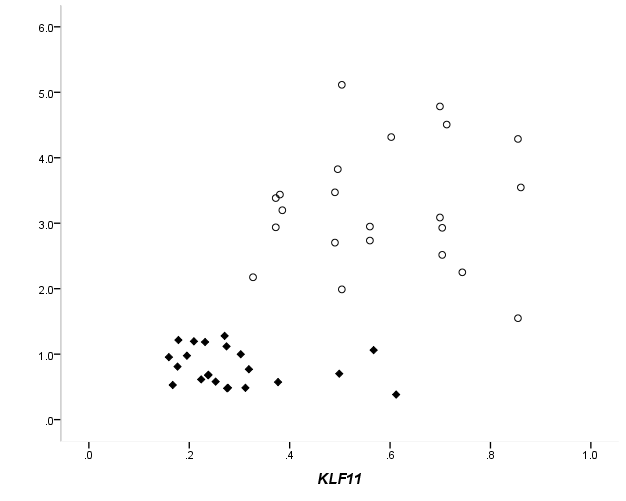 | 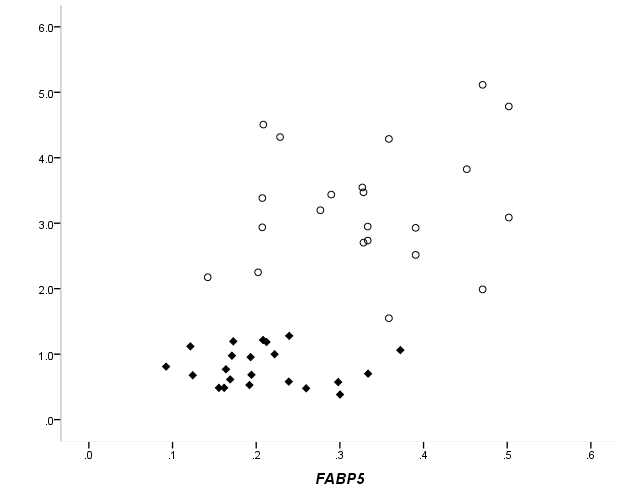 |
| 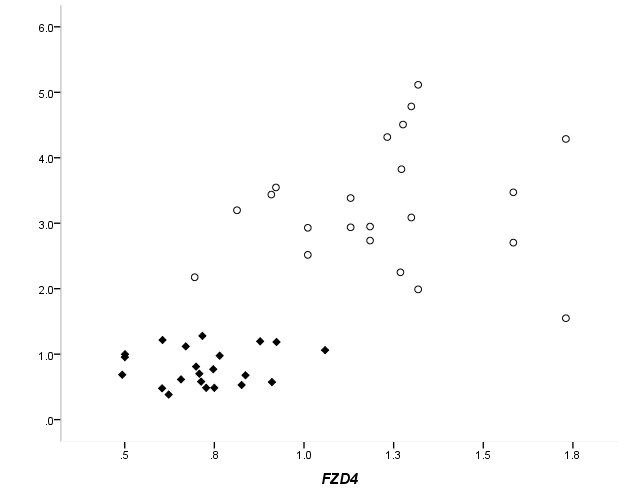 | 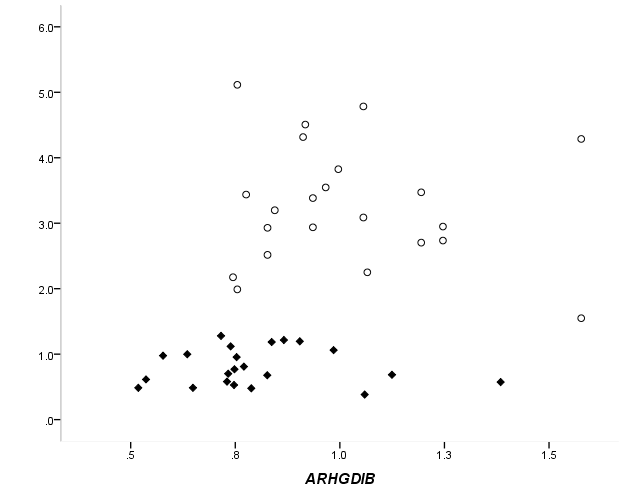 | 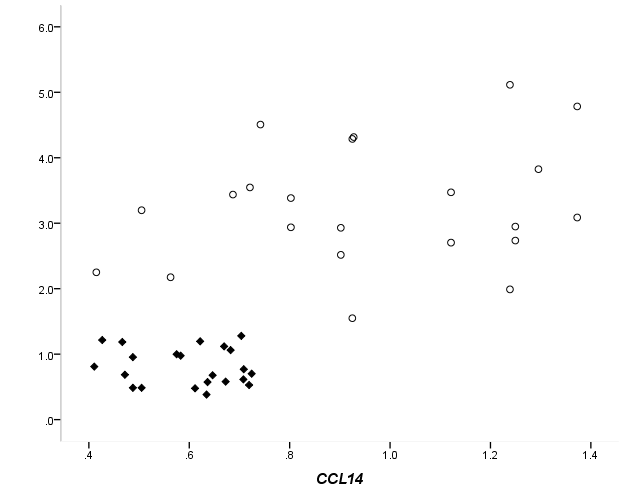 |
| 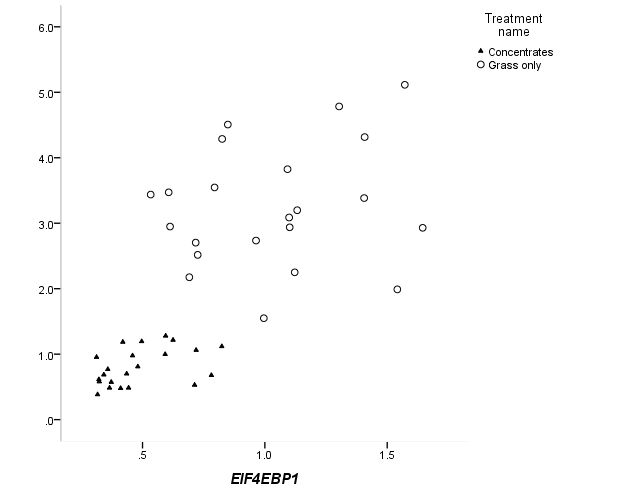 | 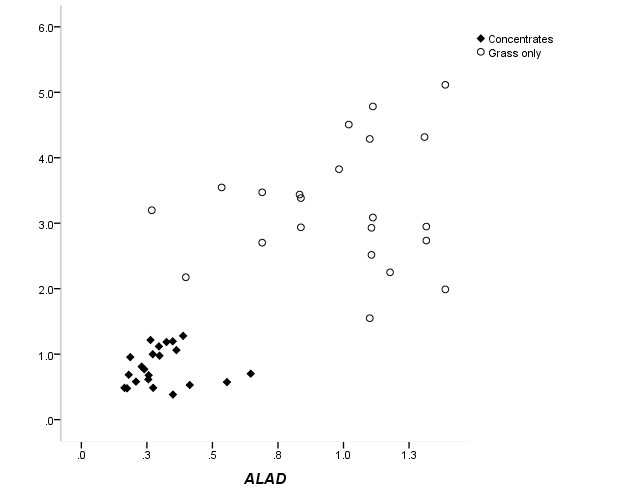 |  |

| 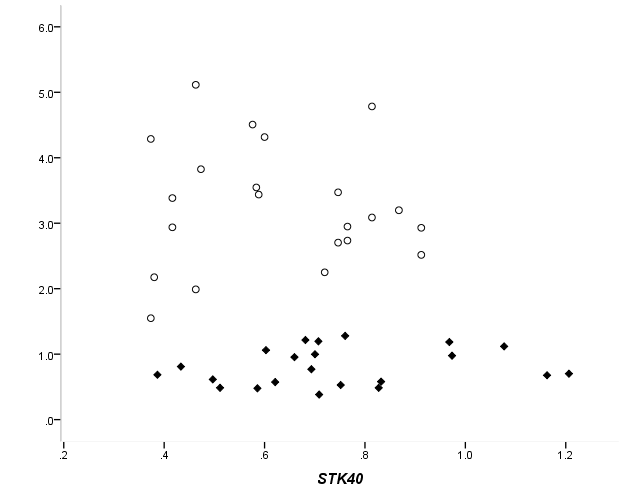 | 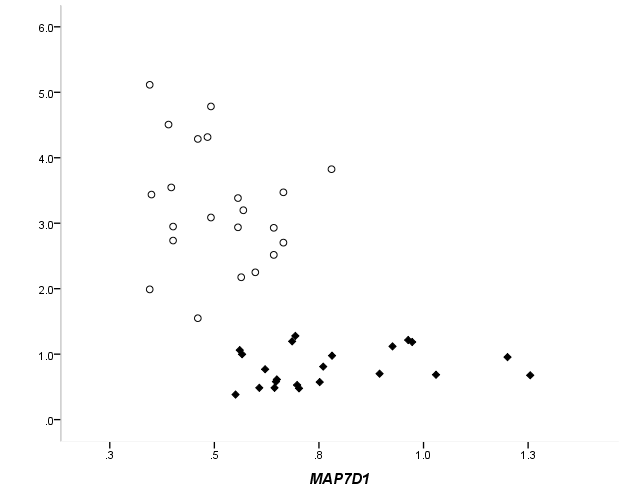 | 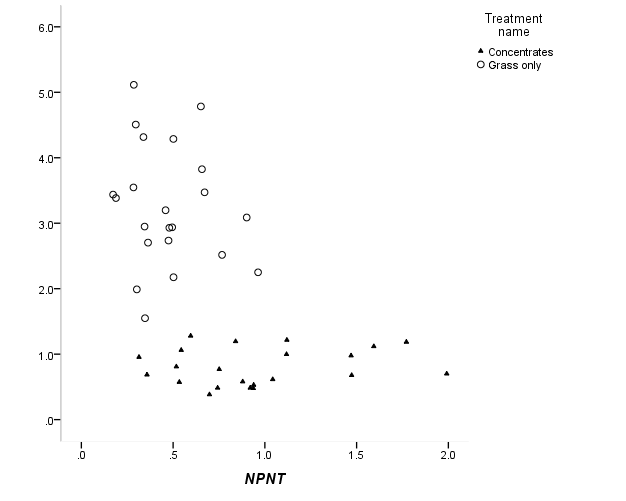 |
| --- | --- | --- |
| 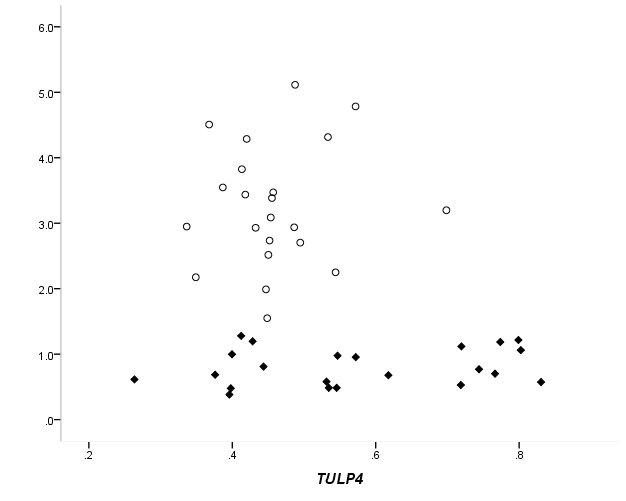 | 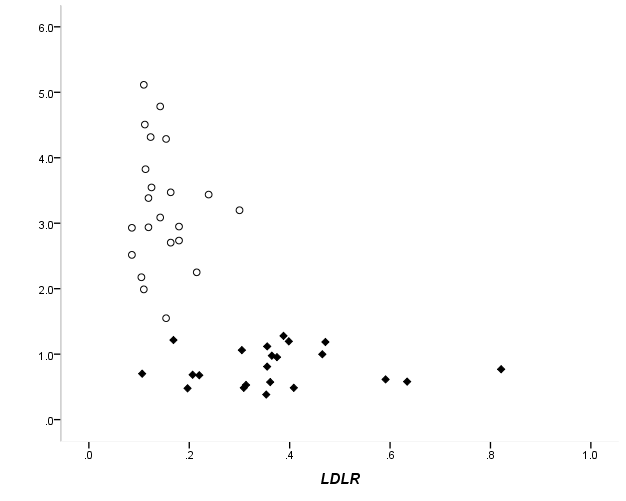 |  |

Figure S3 Scatter plots of Omega 3 fatty acid concentrations (g/100g fatty acid methyl ester) versus normalised relative quantities for DE genes, for the grass-fed (n=22) and concentrate-fed animals (n= 22).

**◦** Grass-Fed animals

⬩ Concentrate fed animals
