# Supplementary material for: The application of transcriptomic data in the authentication of beef derived from contrasting production systems
Source: BMC Genomics. 2016 Sep 21;17:746. doi: 10.1186/s12864-016-2851-7 (PMC5031250; doi:10.1186/s12864-016-2851-7)
Supplement: Additional file 4: Figure S4. — Heat map and R2 values for selected DE genes derived from correlation matrix of QPCR data (n = 44). (DOCX 21 kb) [file 12864_2016_2851_MOESM4_ESM.docx]

Figure S4 Pearson correlation (R ^2^) of normalised relative quantities generated by qPCR for total dataset (n=44)

| Gene symbol | Up-regulated in Grass-fed group | | | | | | | | | | | | Down-regulated in Grass-fed group | | | | | |
| --- | --- | --- | --- | --- | --- | --- | --- | --- | --- | --- | --- | --- | --- | --- | --- | --- | --- | --- |
|  | *CPT1B* | *PLIN5* | *FYN* | *ABCA1* | *KLF11* | *FABP5* | *FZD4* | *ARHGDIB* | *EIF4EBP1* | *ALAD* | *FCGRT* | *CCL14* | *STK40* | *ST6GALNAC4* | *MAP7D1* | *NPNT1* | *TULP1* | *LDLR* |
| *CPT1B* | 1 |  |  |  |  |  |  |  |  |  |  |  |  |  |  |  |  |  |
| *PLIN5* | **0.77** | 1 |  |  |  |  |  |  |  |  |  |  |  |  |  |  |  |  |
| *FYN* | **0.47** | **0.43** | 1 |  |  |  |  |  |  |  |  |  |  |  |  |  |  |  |
| *ABCA1* | **0.64** | **0.57** | **0.32** | 1 |  |  |  |  |  |  |  |  |  |  |  |  |  |  |
| *KLF11* | **0.81** | **0.66** | **0.36** | **0.74** | 1 |  |  |  |  |  |  |  |  |  |  |  |  |  |
| *FABP5* | **0.74** | **0.53** | **0.38** | **0.55** | **0.66** | 1 |  |  |  |  |  |  |  |  |  |  |  |  |
| *FZD4* | **0.64** | **0.78** | **0.51** | **0.64** | **0.69** | **0.47** | 1 |  |  |  |  |  |  |  |  |  |  |  |
| *ARHGDIB* | **0.63** | **0.58** | 0.26 | **0.62** | **0.53** | **0.38** | **0.58** | 1 |  |  |  |  |  |  |  |  |  |  |
| *EIF4EBP1* | **0.59** | **0.53** | **0.6** | **0.35** | **0.55** | **0.59** | **0.55** | 0.11 | 1 |  |  |  |  |  |  |  |  |  |
| *ALAD* | **0.72** | **0.77** | **0.57** | **0.56** | **0.68** | **0.45** | **0.8** | **0.51** | **0.73** | 1 |  |  |  |  |  |  |  |  |
| *FCGRT* | **0.72** | **0.75** | 0.27 | **0.56** | **0.68** | **0.52** | **0.67** | **0.67** | **0.52** | **0.79** | 1 |  |  |  |  |  |  |  |
| *CCL14* | **0.64** | **0.65** | **0.47** | **0.45** | **0.51** | **0.63** | **0.68** | **0.47** | **0.56** | **0.76** | **0.72** | 1 |  |  |  |  |  |  |
| *STK40* | -0.14 | -0.09 | -0.22 | -0.18 | -0.18 | -0.23 | -0.17 | -0.27 | -0.04 | -0.11 | 0.01 | **-0.09** | 1 |  |  |  |  |  |
| *ST6GALNAC4* | **-0.34** | **-0.25** | **-0.31** | **-0.41** | **-0.51** | **-0.43** | **-0.41** | -0.15 | -0.45 | **-0.4** | -0.18 | **-0.38** | **0.55** | 1 |  |  |  |  |
| *MAP7D1* | **-0.5** | **-0.48** | **-0.38** | **-0.46** | **-0.63** | **-0.46** | **-0.52** | **-0.24** | **-0.39** | **-0.54** | **-0.44** | **-0.45** | **0.45** | **0.6** | 1 |  |  |  |
| *NPNT1* | **-0.45** | **-0.43** | **-0.34** | **-0.32** | **-0.37** | **-0.36** | **-0.35** | **-0.42** | **-0.33** | **-0.38** | **-0.36** | **-0.36** | **0.7** | **0.54** | **0.56** | 1 |  |  |
| *TULP1* | -0.08 | -0.08 | -0.08 | -0.12 | -0.26 | -0.13 | -0.13 | -0.08 | -0.12 | -0.23 | -0.12 | -0.25 | **0.5** | **0.56** | **0.42** | **0.46** | 1 |  |
| *LDLR* | **-0.6** | **-0.44** | **-0.35** | **-0.55** | **-0.54** | **-0.43** | **-0.47** | **-0.44** | **-0.63** | **-0.56** | **-0.55** | **-0.39** | 0.19 | 0.27 | 0.22 | 0.29 | 0.24 | 1 |

*Significant correlations (P<0.05) are highlighted in bold
