# Supplementary material for: The application of transcriptomic data in the authentication of beef derived from contrasting production systems
Source: BMC Genomics. 2016 Sep 21;17:746. doi: 10.1186/s12864-016-2851-7 (PMC5031250; doi:10.1186/s12864-016-2851-7)
Supplement: Additional file 5: Figure S5. — Outputs from cScan analysis for human transcripts a) up-regulated and b) down-regulated in the outdoor/pasture-fed group. Table S1. Summary of histone modifications identified on human paralogues and regulatory motifs on the upstream region of the bovine sequence of DE genes. Table S2. Human Ensembl IDs related to gene names of DE genes (a) up-regulated and (b) down-regulated in the pasture-fed group. (DOCX 210 kb) [file 12864_2016_2851_MOESM5_ESM.docx]

Figure S5 Outputs from cScan analysis for human transcripts a) up-regulated and b) down-regulated in the outdoor/pasture-fed group.

a)


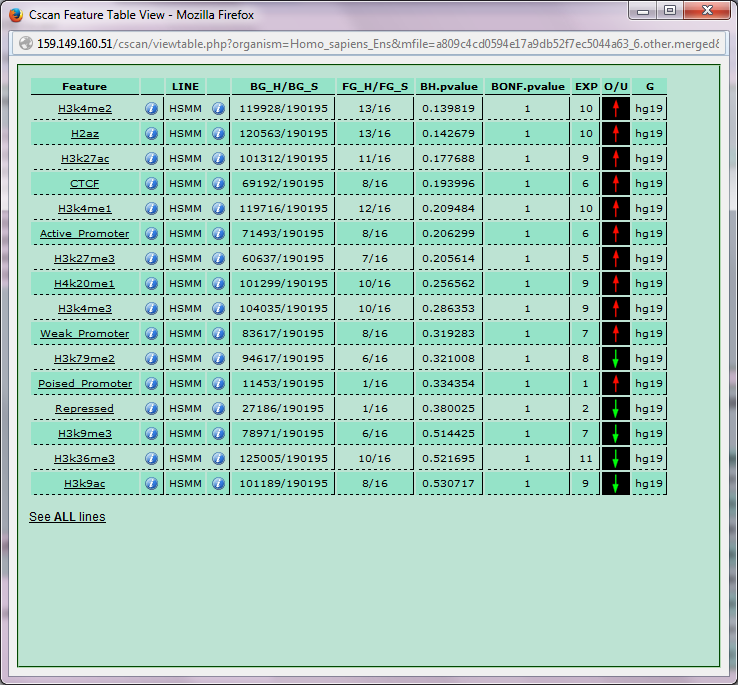


b)


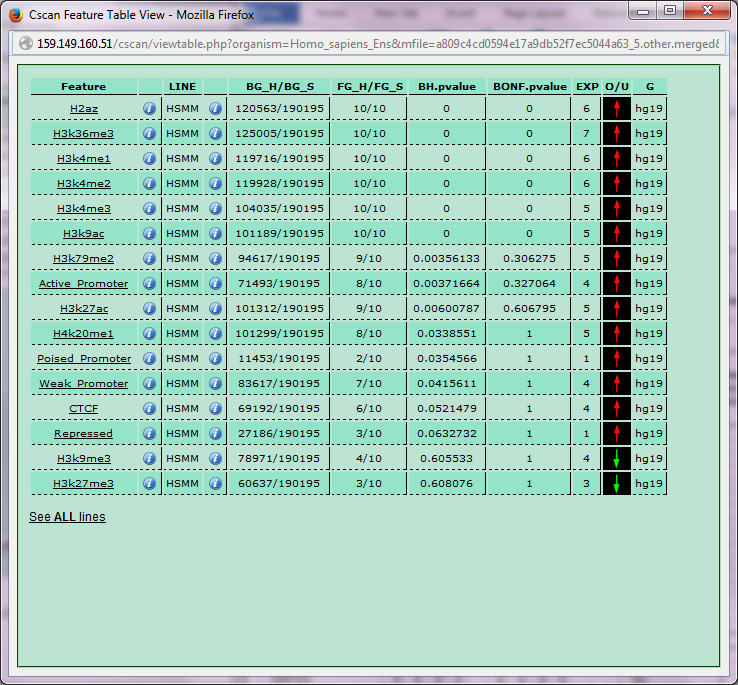


Table S2 Summary of histone modifications identified on human paralogues and regulatory motifs on the upstream region of the bovine sequence of DE genes

| **Gene Name** | **Promoters of human orthologes which are positive for histone markers in muscle cell line HSMM (-1000 bp/TrReg )** | | | | | | | | | | | **CpG Islands in promoter of Bovine sequence**  **(UCSC)** | **Putative selected TFBS motifs in bovine promoter (-1000bp upstream ) identified in Jasper vertebrate database** | | | |
| --- | --- | --- | --- | --- | --- | --- | --- | --- | --- | --- | --- | --- | --- | --- | --- | --- |
|  | **H3K9ac** | **H2az** | **H3k79me2** | **H3k36me3** | **H3k4me1** | **H3k4me2** | **H3K4me3** | **H4k20me1** | **H3K27ac** | **H3K27me3** | **H9K9me3** |  | **PPARδ** | **CEBP (***a, β***)** | **SREBP (-1,-2)** | **GATA (-1,-2,-3)** |
| *CPT1B* |  | **√** |  |  | **√** | **√** |  | **√** |  | **√** | **√** | Yes | 4 | 5 | 5 | 2 |
| *FYN* | **√** | **√** | **√** | **√** | **√** | **√** | **√** | **√** | **√** |  | **√** | No | 1 | 27 | 6 | 16 |
| *PLIN5* |  | **√** |  |  | **√** | **√** |  | **√** |  | **√** |  | Yes | 1 | 20 | 1 | 11 |
| *ABCA1* | **√** | **√** |  | **√** | **√** | **√** | **√** | **√** | **√** | **√** | **√** | Yes | 2 | 21 | 4 | 10 |
| *KLF11* |  | **√** |  | **√** |  | **√** | **√** | **√** | **√** | **√** | **√** | Yes | 4 | 6 | 5 | 2 |
| *FABP5* | **√** | **√** | **√** | **√** | **√** | **√** | **√** |  | **√** | **√** | **√** | Yes | 0 | 11 | 6 | 9 |
| *FZD4* | **√** | **√** |  | **√** | **√** | **√** | **√** |  | **√** |  |  | Yes | 2 | 16 | 6 | 13 |
| *ARHGDIB* |  | **√** |  |  | **√** |  | **√** |  | **√** | **√** |  | No | 0 | 24 | 5 | 30 |
| *EIF4EBP1* |  | **√** | **√** | **√** | **√** | **√** |  | **√** | **√** |  |  | Not available | 1 | 9 | 6 | 14 |
| *ALAD* | **√** | **√** | **√** | **√** | **√** | **√** | **√** | **√** | **√** |  |  | Yes | 3 | 30 | 9 | 24 |
| *FCGRT* | **√** | **√** |  | **√** | **√** | **√** | **√** |  | **√** |  |  | Yes | 3 | 29 | 6 | 30 |
| *CCL14* |  |  |  |  |  |  |  |  |  | **√** |  | No | 5 | 18 | 4 | 18 |
| *BREH1* |  |  |  |  |  |  |  |  |  |  |  | Yes | 1 | 17 | 11 | 25 |
| *RNF149* | **√** | **√** | **√** | **√** | **√** | **√** | **√** | **√** | **√** |  |  | Not available | 4 | 23 | 4 | 12 |
| *ACSL3* | **√** | **√** | **√** | **√** | **√** | **√** | **√** | **√** | **√** |  | **√** | Yes | 3 | 20 | 3 | 13 |
| *GPIHBP1* |  |  |  |  |  |  |  | **√** |  |  |  | Not available | 4 | 9 | 7 | 10 |
| *Significant (P<0.05)* | **ns** | ns | ns | ns | ns | ns | ns | ns | ns | ns | ns |  |  |  |  |  |
| *STK40* | **√** | **√** | **√** | **√** | **√** | **√** | **√** | **√** | **√** |  |  | Yes | 2 | 5 | 6 | 16 |
| *ST6GALNAC4* | **√** | **√** | **√** | **√** | **√** | **√** | **√** | **√** | **√** |  |  | Yes | 0 | 9 | 4 | 17 |
| *MAP7D1* | **√** | **√** | **√** | **√** | **√** | **√** | **√** | **√** | **√** |  |  | Yes | 2 | 14 | 9 | 5 |
| *NPNT* | **√** | **√** |  | **√** | **√** | **√** | **√** | **√** | **√** | **√** | **√** | Not available | 3 | 5 | 0 | 4 |
| *TULP1* | **√** | **√** | **√** | **√** | **√** | **√** | **√** |  | **√** | **√** | **√** | Yes | 2 | 11 | 3 | 20 |
| *LDLR* | **√** | **√** | **√** | **√** | **√** | **√** | **√** | **√** | **√** |  |  | Yes | 3 | 22 | 7 | 21 |
| *MSMO1* | **√** | **√** | **√** | **√** | **√** | **√** | **√** | **√** | **√** |  |  | Yes | 1 | 11 | 9 | 2 |
| *TPCN1* | **√** | **√** | **√** | **√** | **√** | **√** | **√** | **√** | **√** |  |  | Not available | 3 | 16 | 6 | 23 |
| *PDP2* | **√** | **√** | **√** | **√** | **√** | **√** | **√** |  |  |  |  | Not available | 3 | 16 | 2 | 7 |
| *EML1* | **√** | **√** | **√** | **√** | **√** | **√** | **√** | **√** | **√** | **√** | **√** | Not available | 1 | 5 | 8 | 7 |
| *Significant (P<0.05)* | ***** | ***** | ***** | ***** | ***** | ***** | ***** | ***** | ***** | ns | ns |  |  |  |  |  |

Table S3 Human Ensembl IDs related to gene names of DE genes (a) up-regulated and (b) down-regulated in the pasture-fed group.

| Bovine probe ID | **Gene name** | **Human ensemble ID** |
| --- | --- | --- |
| Bt.19423.2.S1_at | *ABCA1* | ENST00000374736 |
| Bt.16916.3.S1_at | *KLF11* | ENST00000535335 |
| Bt.17513.1.A1_at | *PLIN5* | ENST00000381848 |
| Bt.9585.1.S1_at | *ALAD* | ENST00000409155 |
| Bt.1035.1.S1_a_at | *FCGRT* | ENST00000221466 |
| Bt.2359.1.A1_at | *FYN* | ENST00000354650 |
| Bt.22869.1.S2_at | *FABP5* | ENST00000297258 |
| Bt.21113.1.S1_a_at | *CPT1B* | ENST00000360719 |
| Bt.6936.1.S1_at | *CCL14* | ENST00000536149 |
| Bt.1739.2.S1_at | *FZD4* | ENST00000531380 |
| Bt.19795.1.S1_at | *CES1 (BREH1)* | ENST00000422046 |
| Bt.6434.2.S1_at | *RNF149* | ENST00000295317 |
| Bt.19850.2.S1_at | *ACSL3* | ENST00000357430 |
| Bt.4757.1.S1_at | *ARHGDIB* | ENST00000228945 |
| Bt.5389.1.S1_at | *EIF4EBP1* | ENST00000520657 |
| Bt.26962.1.S1_at | *GPIHBP1* | ENST00000330824 |
|  |  |  |
| Bt.2392.1.S1_at | *ST6GALNAC4* | ENST00000335791 |
| Bt.23212.1.S1_at | *MSMO1* | ENST00000261507 |
| Bt.4688.1.S1_a_at | *TPCN1* | ENST00000335509 |
| Bt.13526.1.S1_at | *PDP2* | ENST00000311765 |
| Bt.16265.1.S1_at | *EML1* | ENST00000262233 |
| Bt.6394.1.A1_at | *STK40* | ENST00000373129 |
| Bt.3562.1.S1_at | *LDLR* | ENST00000535915 |
| Bt.11038.1.S1_at | *TULP1* | ENST00000229771 |
| Bt.20458.1.S1_at | *MAP7D1* | ENST00000373151 |
| Bt.7393.1.S1_at | *NPNT* | ENST00000427316 |
