# Supplementary material for: The application of transcriptomic data in the authentication of beef derived from contrasting production systems
Source: BMC Genomics. 2016 Sep 21;17:746. doi: 10.1186/s12864-016-2851-7 (PMC5031250; doi:10.1186/s12864-016-2851-7)

Figure S6 Boxplot of unnormalised log intensity values.


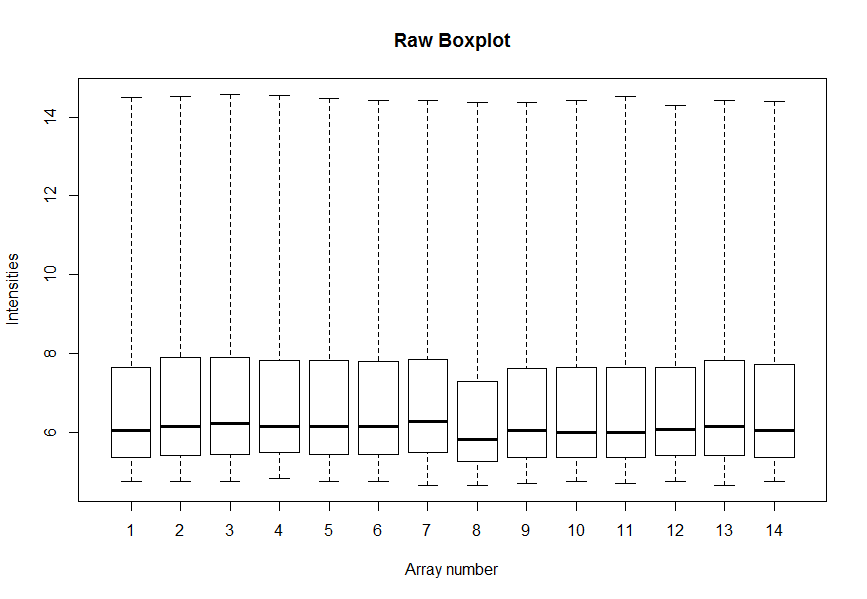


Figure S7 Boxplot of normalised log intensity values.


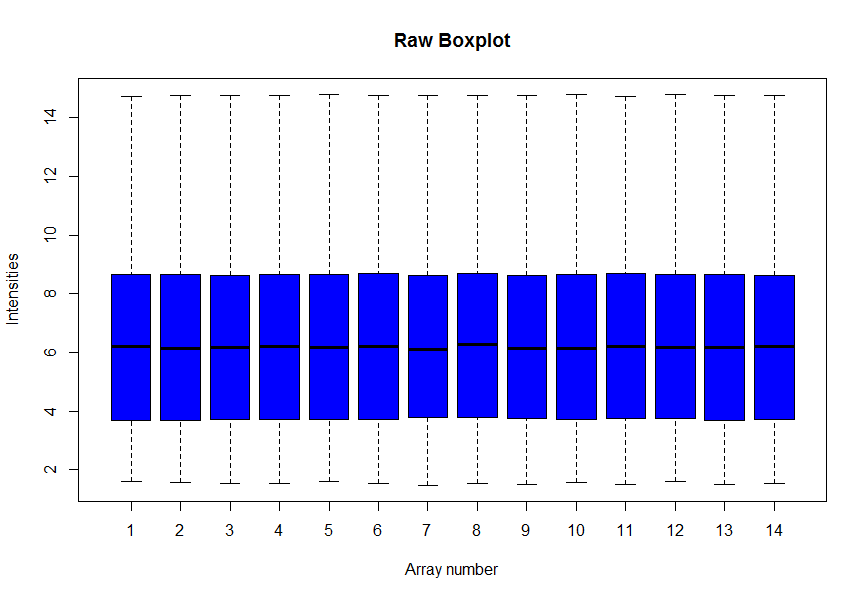


Figure S8 MA-plots for un-normalised data.


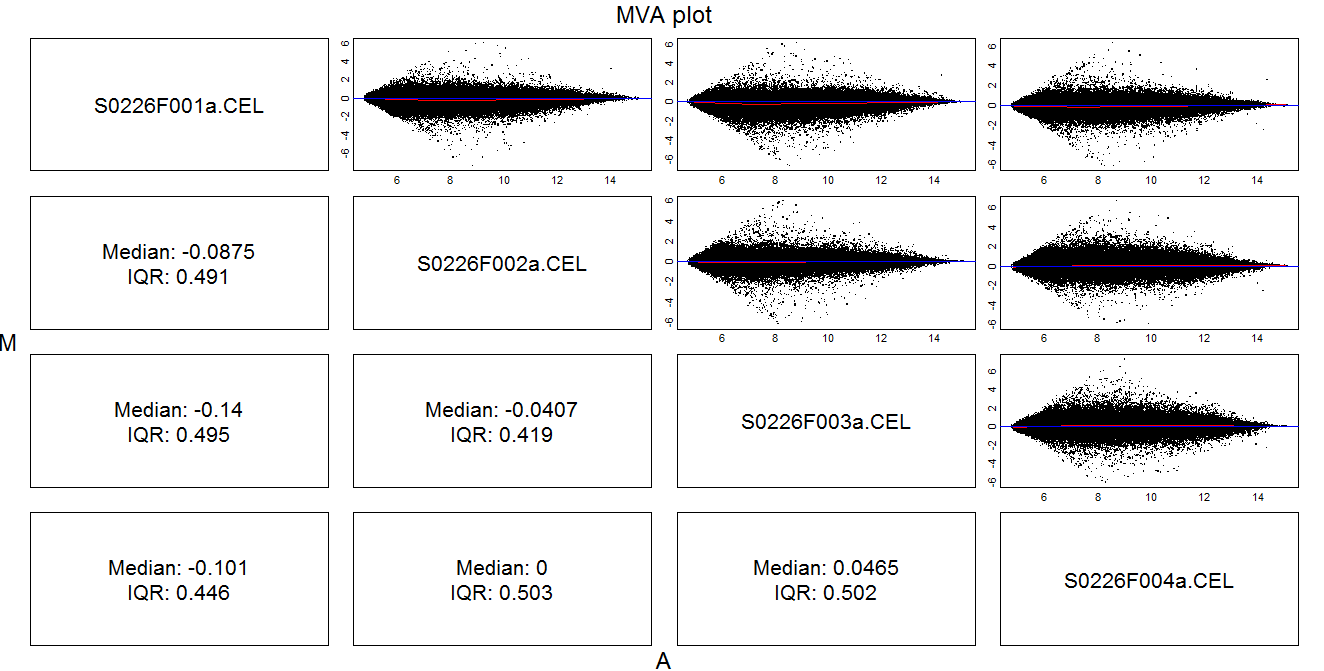


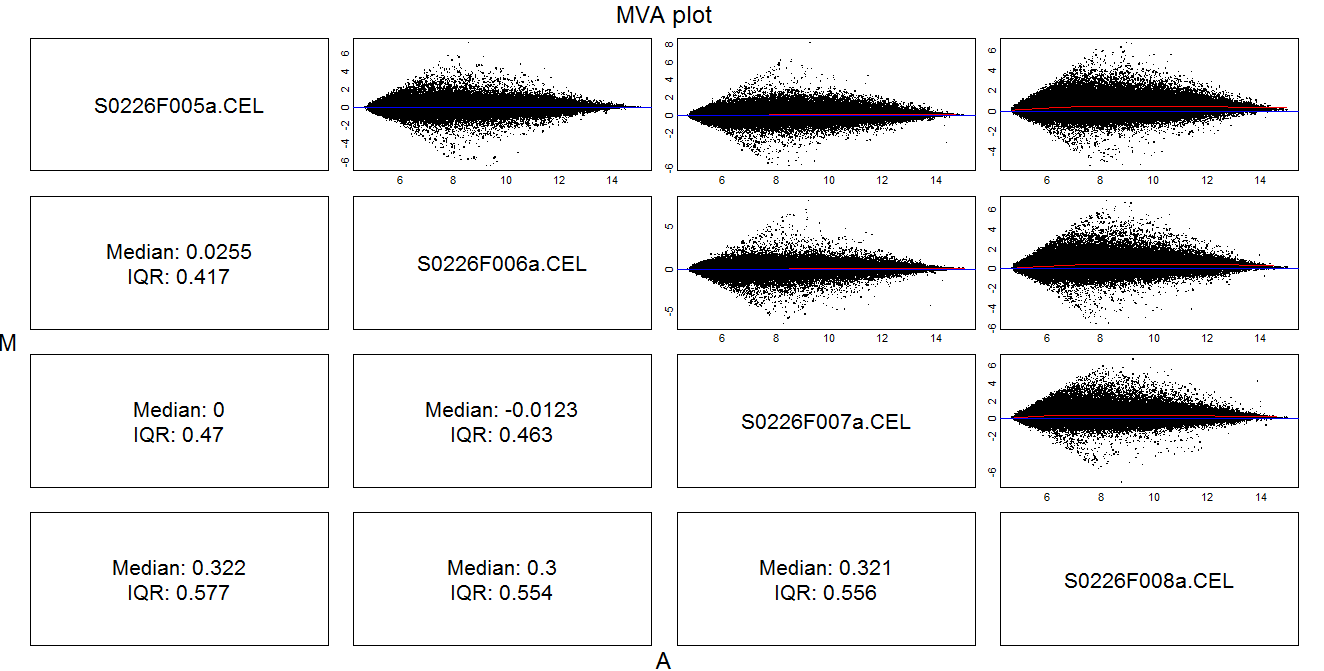


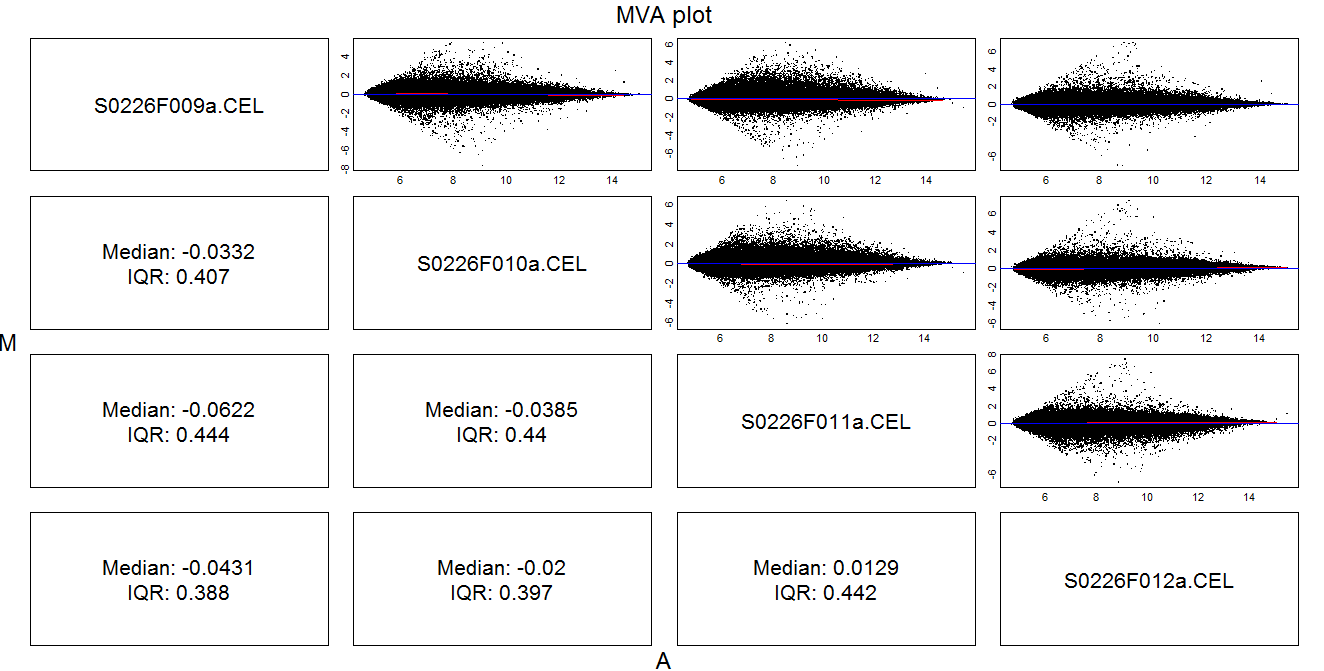


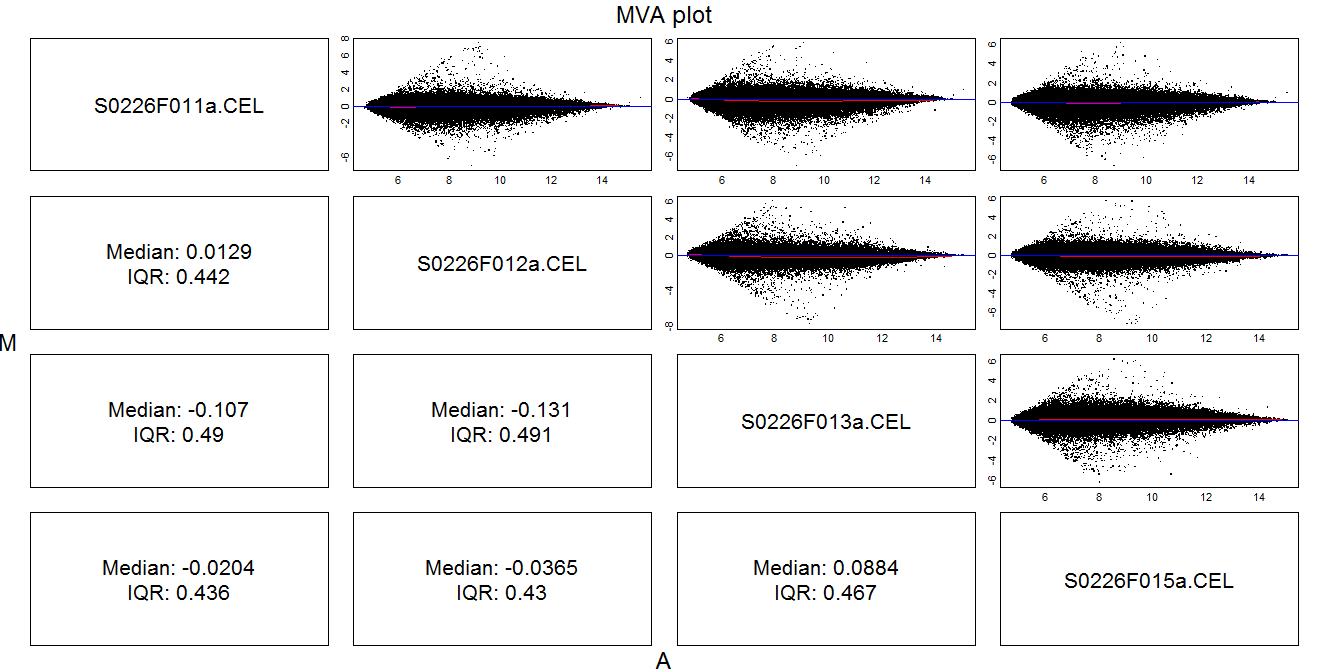


Figure S9 MA-plots for normalised data.


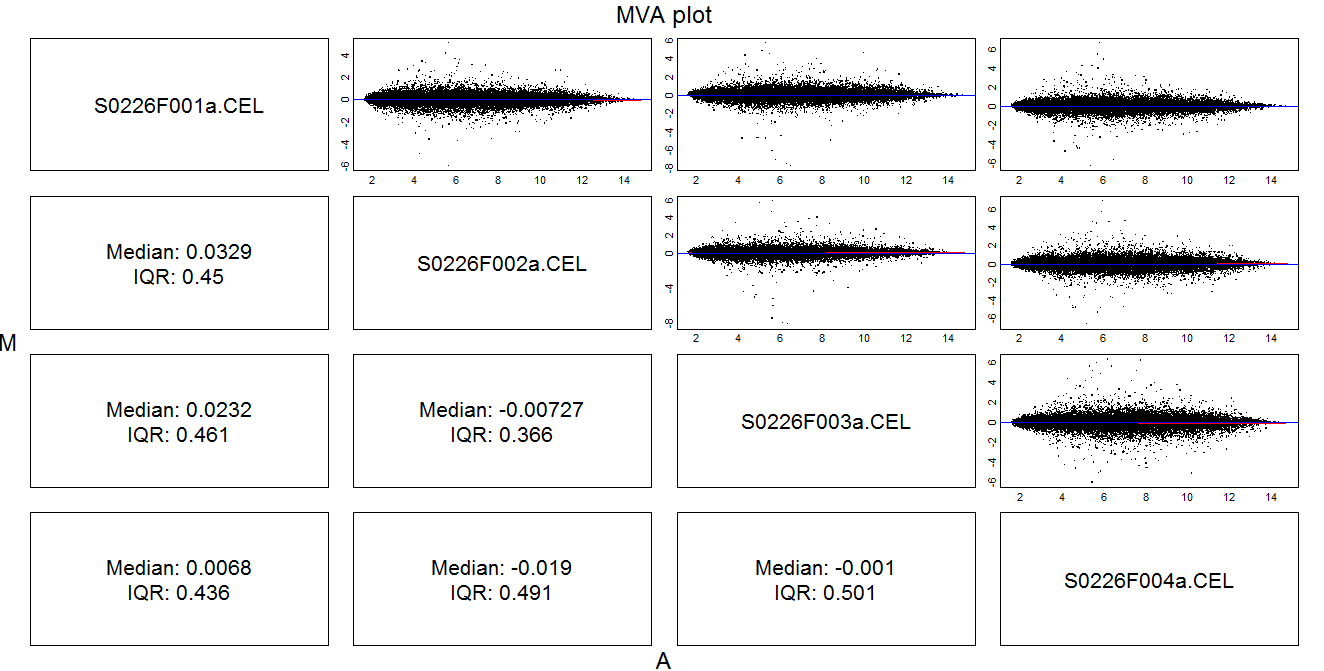


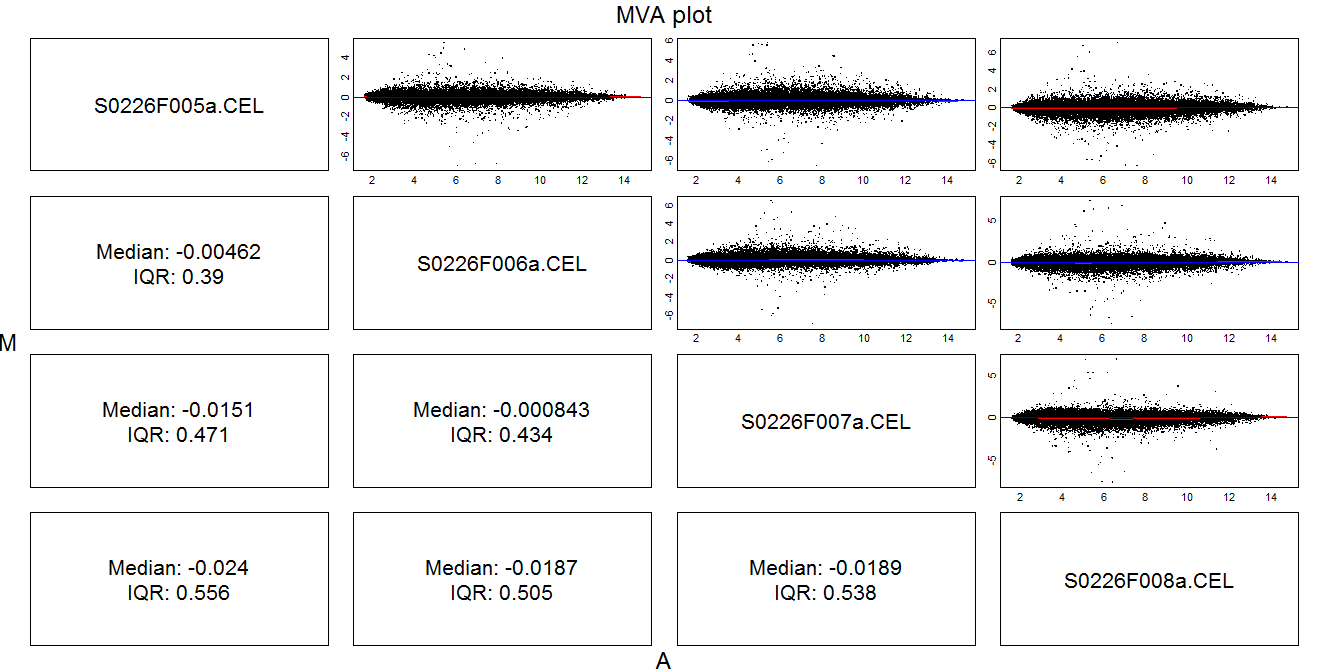


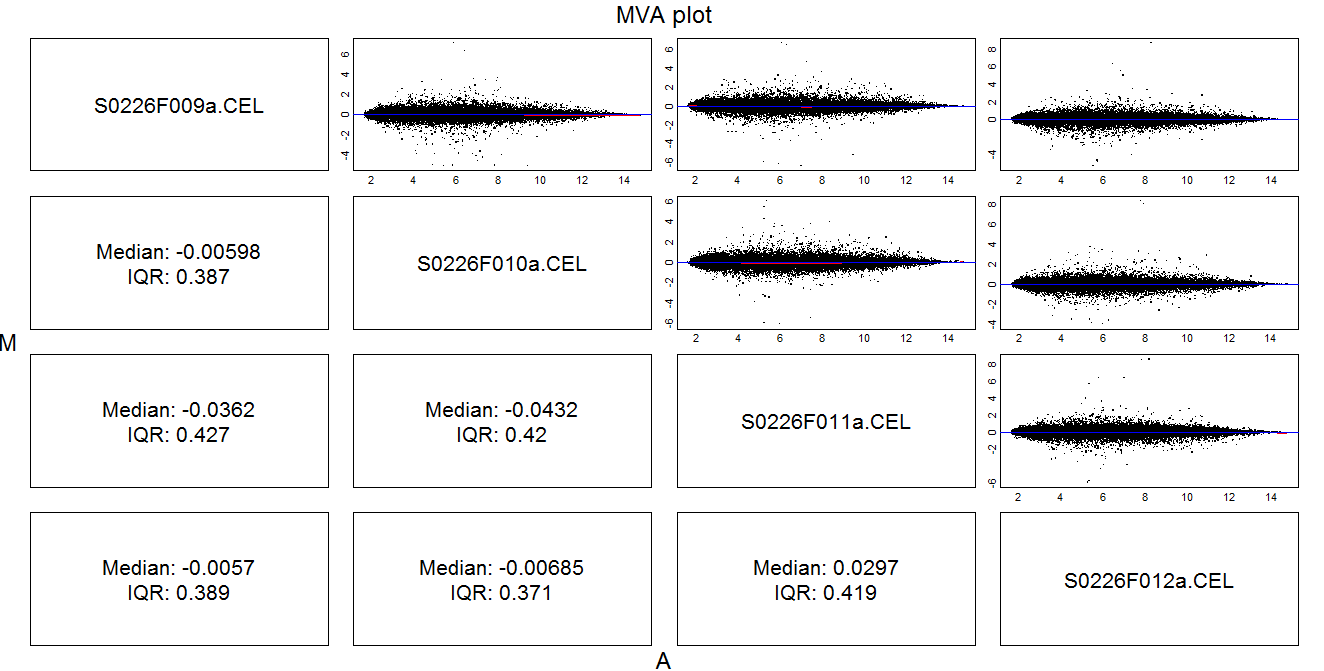


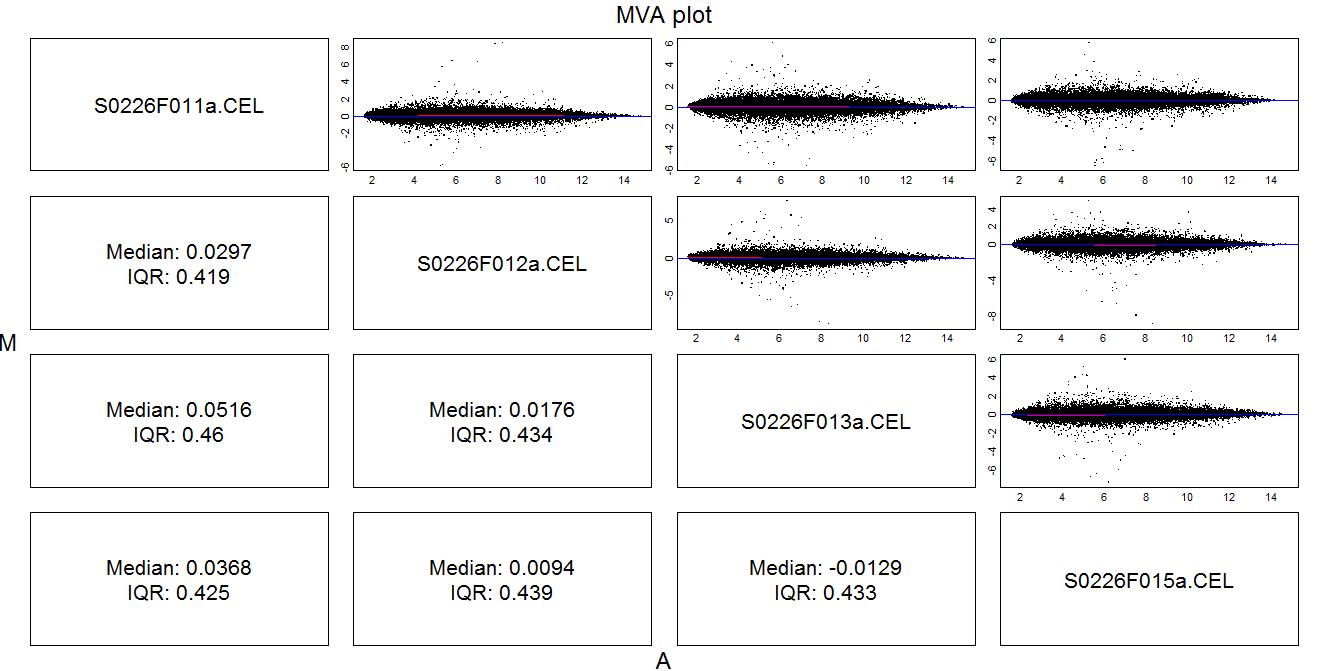


Figure S10 RNA degradation analysis.


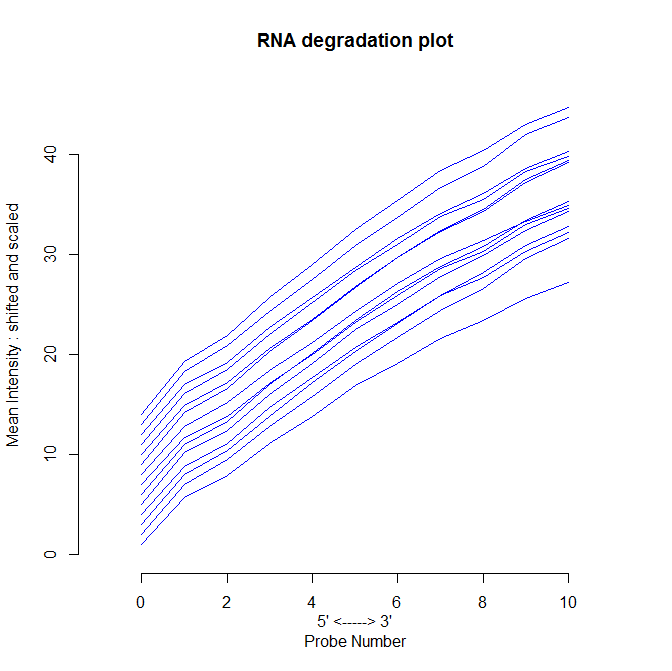


Figure S11 Plots histogram of PM intensities for all 14 arrays.


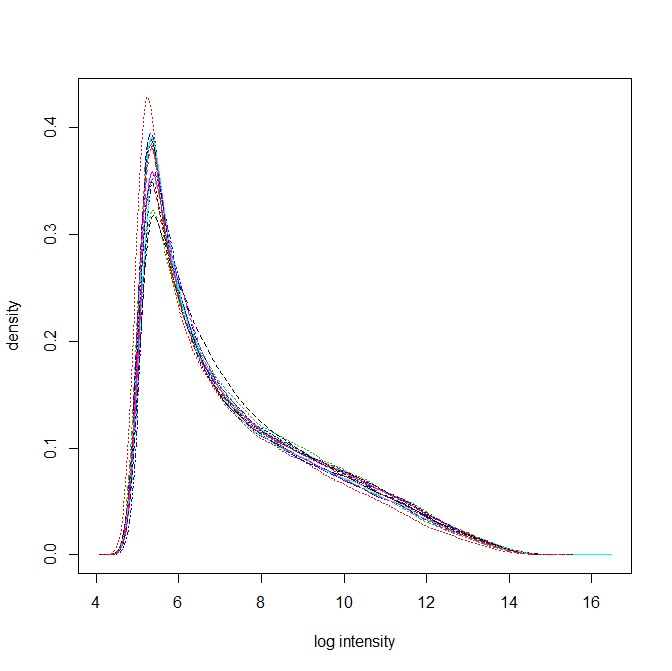

Supplement: Additional file 7: Figure S6. — Boxplot of unnormalised log intensity values. Figure S7. Boxplot of normalised log intensity values. Figure S8. MA-plots for un-normalised data. Figure S9. MA-plots for normalised data. Figure S10. RNA degradation analysis. Figure S11. Histogram of PM intensities for all 14 arrays. (DOCX 532 kb) [file 12864_2016_2851_MOESM7_ESM.docx]
