# Supplementary material for: The application of transcriptomic data in the authentication of beef derived from contrasting production systems
Source: BMC Genomics. 2016 Sep 21;17:746. doi: 10.1186/s12864-016-2851-7 (PMC5031250; doi:10.1186/s12864-016-2851-7)
Supplement: Additional file 8: Table S3. — Primer pairs used for QPCR on DE genes. (DOCX 16 kb) [file 12864_2016_2851_MOESM8_ESM.docx]

| **Gene Name** | **Primer sequences 5'-3'** |
| --- | --- |
| *CPT1B* | F:TGGTCGTGGTTATGGCAACA  R:TAATAGACCAGGCCCATGGAA |
| *FYN* | F:CGCCGACTTCGGATTGG  R:GCGCCTTGTCTTGCTGTGT |
| *ABCA1* | F:CCGGCAGCGTCGAGTTAC  R:GATCTTATTCGTCCTCTCCACATTG |
| *KLF11* | F:GAACCCAGGCAAGAGAATGTG  R:AAAACCATTCCCAGGCTTGA |
| *FABP5* | F:AGTTTCTGTAGGCACTGGGCTAA  R:AATGGTACTAGCTCCCCAAGGATT |
| *FZD4* | F:TTCATCTCCACCGCCTTCA  R:GGGTAGGAAAACCTGGAAGAATC |
| *ARHGDIB* | F:CTGGATTCTCCTGGTGGTTACTG  R:TTGGCAGGGAACGTGATGAT |
| *EIF4EBP1* | F:GGCGGCACGCTCTTCA  R:AGGAACTTCCGGTCATAGATGATC |
| *ALAD* | F:GCCCCGTCGGACATGA  R:ATCCATGTGCCATCAGAGCTT |
| *FCGRT* | F:CCAGCAACTGCCGATCATC  R:CATGAAAAGGACCTGAGGACATT |
| *CCL14* | F:GTGCCAACCCCCGTGAT  R:GGGTCACTCCTCCAGTTCCTT |
| *STK40* | F:CCCTGGGCGTGGTACTGTT  R:TGCGGGATGCTGTCGTAG |
| *ST6GALNAC4* | F:CATCACCCTCAGTGCCTTAC  R:TGCGCCAGGTACATTTGACA |
| *MAP7D1* | F:TCCGTGTCGGCAGTAAACCT  R:GACTTTGAGAGCCGCTTGTTG |
| *NPNT1* | F:TGATCGACAGGCCCACTTCT  R:GGCTCTGGTATTGGCTTTGG |
| *TULP1* | F:GGCCATCTCCCCGCTATTAC  R:AATTTACCCACCAAAGAATCAGGT |
| *LDLR* | F:TTTCCAGTTCAGGGTCGAACAC  R:CCAGAACGGAAAGTAGTGCAAA |
| *RPLP0* | F:CAACCCTGAAGTGCTTGACAT  R:AGGCAGATGGATCAGCCA |
| *YWHAZ* | F:GCATCCCACAGACTATTTCC  R:GCAAAGACAATGACAGACCA |

Additional file 8: Table S3: Primer pairs used for QPCR on DE genes.
